# Carbohydrate Structure Database: tools for statistical analysis of bacterial, plant and fungal glycomes

**DOI:** 10.1093/database/bav073

**Published:** 2015-09-03

**Authors:** K.S. Egorova, A.N. Kondakova, Ph.V. Toukach

**Affiliations:** N.D. Zelinsky Institute of Organic Chemistry, Russian Academy of Sciences, Leninskiy prospect 47, 119991 Moscow, Russia

## Abstract

Carbohydrates are biological blocks participating in diverse and crucial processes both at cellular and organism levels. They protect individual cells, establish intracellular interactions, take part in the immune reaction and participate in many other processes. Glycosylation is considered as one of the most important modifications of proteins and other biologically active molecules. Still, the data on the enzymatic machinery involved in the carbohydrate synthesis and processing are scattered, and the advance on its study is hindered by the vast bulk of accumulated genetic information not supported by any experimental evidences for functions of proteins that are encoded by these genes. In this article, we present novel instruments for statistical analysis of glycomes in taxa. These tools may be helpful for investigating carbohydrate-related enzymatic activities in various groups of organisms and for comparison of their carbohydrate content. The instruments are developed on the Carbohydrate Structure Database (CSDB) platform and are available freely on the CSDB web-site at http://csdb.glycoscience.ru.

**Database URL**: http://csdb.glycoscience.ru

## Introduction

In molecular biology, carbohydrates had long been shaded by other biological ‘building units’, such as proteins. It is established knowledge that cells of prokaryotic and eukaryotic organisms possess the cell wall, which contains peptidoglycan, or murein (bacteria) (1, 2); branched beta-glucans linked to chitin (fungi) (3) or cellulose, hemicelluloses and pectin (plants) (4). However, only when the importance of protein glycosylation began to emerge, the fundamental role of carbohydrates in numerous processes, such as development of immunological memory, was established.

Glycosylation is one of major post-translational modifications of proteins and is found in specimens from almost all domains of life, from bacteria to mammals. Glycan patterns can affect intra- and intermolecular interactions, as well as cell-cell contacts and adhesion (5). Now we know that bacterial and fungal carbohydrates, which are per se weakly immunogenic, as a part of glycoproteins or lipopolysaccharides (LPSs) can trigger a carbohydrate-specific T cell immune reaction resulting in antibody production and immunological memory development (6–8). Diverse carbohydrates allow pathogenic microorganisms to find ways to bypass the immune response of a host organism. Thus, many infectious bacteria and viruses expose carbohydrates on their surface, and the structure of these carbohydrates resembles closely that one of the molecules, which are present in surface glycoproteins and glycolipids of host cells (6).

Carbohydrates of numerous pathogenic bacteria attract attention as candidates for vaccines (9). Bacterial cell surface capsular polysaccharides and LPSs can be utilized as antigens for obtaining antibodies against the corresponding infection, and glycoconjugate vaccines seem to be effective in developing protective immune reactions. When n- and o-glycosylation of bacterial proteins was discovered, new perspectives in biotechnology have opened (5). Therefore, genetic and proteomic information on enzymatic apparatus involved in the synthesis of bacterial carbohydrates and glycoconjugates is of particular importance.

Bacteria are not the only organisms whose carbohydrate structures present interest for fundamental science and medicine. In fungi, glycoproteins and glycolipids show diversity and complexity. For example, *Cryptococcus neoformans* surrounds its cell wall with a polysaccharide capsule that defines its virulence (10). Pathogenic fungi possess glycosyltransferases (GTs) missing from humans and therefore presenting potential pharmaceutical targets that allow avoiding host toxicity typical for many antifungal substances.

Cell walls of bacteria and fungi are known to be dynamic structures, which provide both protection from and interaction with the environment. Cell walls of pathogenic and commensal microorganisms are recognized by host receptors, such as nucleotide oligomerization domain proteins, peptidoglycan recognition proteins, Toll-like receptors and C-type lectin receptors, which, in turn, mediate the organism reaction to the invasion (11). In order to escape the host immune response, microorganisms use several tricks by modifying their cell walls (11). Thus, the success and survival of these bacteria and fungi depend on their ability to synthesize particular carbohydrate-containing structures. There is a whole complex of enzymes, called carbohydrate-active enzymes (CAZy), which mediate synthesis, assembly and processing of carbohydrate-containing compounds. The CAZy database combines numerous families of glycoside hydrolases, GTs, polysaccharide lyases and carbohydrate esterases, as well as carbohydrate-binding modules (12, 13). Outer carbohydrates are one of the most variable characteristics of microbial cells (11, 14), and the glycome of such bacterial and fungal species should reflect mechanisms of their interaction with the host, as well as their genetic constitution (in terms of CAZy-encoding genes).

Plants constitute another group of organisms whose carbohydrates are important for medicine. Numerous plant bioactive low-molecular weight products demonstrate specific patterns of glycosylation (15). Sugar moieties may modulate biological activity of these compounds, and where existing chemical technologies do not allow implementing effective specific glycosylation of complex molecules, enzymatic procedures can be used. Therefore, information on activity and selectivity of plant carbohydrates and GTs is demanded.

From 2005, we have been developing and maintaining the Carbohydrate Structure Database (CSDB) intended for provision of latest data on structures, bibliography, taxonomy, nuclear magnetic resonance (NMR) spectroscopy and other information regarding natural carbohydrates. CSDB includes Bacterial (BCSDB) and Plant and Fungal (PFCSDB) parts, which deposit glycans and glycoconjugates found in prokaryotes, plants and fungi (16, 17). These databases are freely available at http://csdb.glycoscience.ru.

This article presents novel CSDB instruments for statistical analysis of structural feature distribution in taxonomic groups. Completeness of the CSDB coverage on bacterial carbohydrates suggests that such analysis of bacterial glycomes would produce statistically significant results, which could be used for deciphering CAZy activities. CSDB data on plants and fungi, though not complete at the moment, also present a possibility of correlating the information on predicted CAZy genes and proteins with the existing carbohydrates. The article offers potential applications of the statistical tools in the modern glycobiological research.

## Results and Discussion

A survey on database content, especially in databases claiming for complete coverage, often provides valuable information for researches. CSDB has several tools, which can be used in statistical studies. Links to them are present in the ‘Extras’ section of the main menu of the Bacterial (BCSDB, http://csdb.glycoscience.ru/bacterial/index.html) and Plant and Fungal (PFCSDB, http://csdb.glycoscience.ru/plant_fungal/index.html) CSDB. The ‘Fragment abundance’ tool calculates the abundance of monomers and dimers found in carbohydrates from specified taxonomic groups, whereas the ‘Coverage statistics’ tool gives statistics on the database coverage for specified groups. The choice of the database is of significant importance: PFCSDB provides data on algae, fungi and plants, whereas BCSDB—on bacteria, archaea and protista. Note that though the groups ‘bacteria’, ‘archaea’ and ‘protista’ are available from PFCSDB (and vice versa), the correct database should be used in each case.

The ‘Taxon clustering’ tool can be launched from either database with the same result. This tool generates distance matrices for mono- or dimeric fragment pools from taxa populated in both databases. Based on these matrices, the taxa are clustered into groups, and corresponding dendrograms are displayed.

When using CSDB, please keep in mind that the term ‘structure’ refers to an oligomeric molecule or a polymer repeating unit built up of residues linked by ester, ether or amide linkages. Every entity, which is attached to the other part of the molecule via these linkages, is considered a distinct residue (including acetic acid, methanol and other monovalent residues). To be classified as a glycan, the structure should have at least one carbohydrate residue. A dimer is a structural fragment built of two residues of any type. More detailed explanation of terms has been published recently (16).

The following sections describe possible applications of the developed tools in scientific practice. More detailed user manual and examples for the fragment abundance and coverage statistics tools will be published elsewhere.

### Interface

#### Fragment abundance

**Figure 1.**
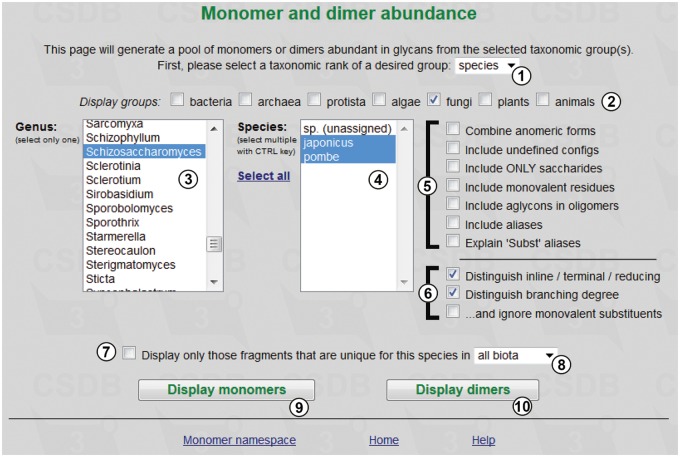
Fragment abundance form.

The tool estimates the abundance of monomers and/or dimers present in glycans from specified taxonomic groups of different ranks. The query form is shown in Figure 1. User can define the source from which the data are gathered, by selecting taxonomic rank ①, domain groups ② and specific taxa ③④. The available ranks are domain, phylum, class, genus, species and strain/subspecies. Figure 1 shows an exemplary query on two species ④ of *Schizosaccharomyces* ③, carbohydrates from which are present in PFCSDB.

The ‘Fragment abundance’ tool provides the following filters ⑤:

- Combine anomeric forms (treats different anomeric forms of the same residue as a single entity); when checked, the resulting table will contain e.g. fragments with the DGlcp residue combining α- and β-anomers; otherwise fragments containing α- and β-anomers of d-glucopyranose (aDGlcp and bDGlcp) will occupy different rows.
- Include undefined configs (underdetermined residues with missing configurations are included into the query scope); when checked, the resulting table will include fragments containing ?Kdop, b?Fucp, ?DGal?, etc.
- Include ONLY saccharides (no monovalent residues, aglycons or aliases are shown). Please note that acetic acid is treated as a separate monovalent residue; thus, with this option checked Ac(1-2)GlcN will not be picked up as a dimer from the structure Gal(1-3)GlcNAc, while Gal(1-3)GlcN will be.
- Include monovalent residues (monovalent substituent residues are included into the query scope); e.g. GlcNAc or Glc-1OMe will be included as Ac(1-2)GlcN or Glc(1-1)Me, accordingly. This and the previous filter are interdependent.
- Include aglycons in oligomers (aglycons are included into the query scope as separate residues);
- Include aliases. Aliases (explained entities missing from the total residue list) and superclasses (LIP, HEX, etc.) are included into the query scope. For example, with this option checked, two dimers—Subst1(1-3)aDGlcp and aDGlcp(1-1)LIP - and three monomers will be picked up from the structure Subst1(1-3)aDGlcp(1-1)LIP // Subst1 = 2,5-diaminopentanoic acid.
- Explain ‘Subst’ aliases (if unchecked, substituents without reserved residue names are displayed as ‘Subst’ and treated together; otherwise they are differentiated). For example, with this option checked the above-mentioned structure will produce the 2,5-diaminopentanoic acid(1-3)aDGlcp dimer; otherwise it will produce the Subst1(1-3)aDGlcp dimer, which will be combined with dimers from other structures possessing Subst1 alias with any explanation.

Checkboxes ⑥ allow distinguishing the position of residues in bigger saccharides and the residue branching degree. The branching degree is a number of substituents, excluding the acceptor residue at the anomeric center (or at another default center in non-sugars). By default, it includes monovalent residues, if they are not ignored by the third checkbox in the group. For example, the branching degree of GlcN in the structure Gal(1-3)GlcNAc(1-2)Man is ‘di-branched’ with monovalent residues counted, or ‘linear’ without monovalent residues.

Checkbox ⑦ allows showing only fragments that are unique for a selected taxon among all biota or kingdom/phylum which it belongs to ⑧. Buttons ⑨ and ⑩ display statistics on monomers and dimers, correspondingly.

**Figure 2.**
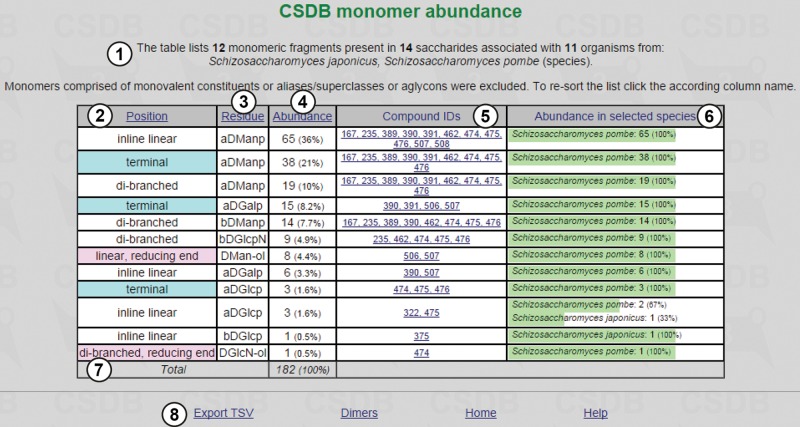
Monomeric composition for *S. japonicus* and *S. pombe*.

In this example, pressing button ⑨ displays the table of monomers present in glycans and glycoconjugates from *Schizosaccharomyces japonicus* and *Schizosaccharomyces pombe* (Figure 2).

Figure 2 shows an overview of fragments, structures and organisms found ①. The table of results is composed of the following columns: position of a residue in the structure ② (if it was checked to be distinguished; terminal residues are shown in cyan, and residues at the reducing end are in pink; the branching degree is also indicated); residue names and configurations ③; abundance ④ (how many times a particular residue occurs in the structures matching the query); compound IDs ⑤ (links to the corresponding compound pages) and abundance (absolute and relative) in selected taxa ⑥. The columns can be sorted by position, residue name or abundance by clicking on column captions ②, ③ or ④. The page contains accessory links, e.g. export of results as tab-separated values for copy-pasting into other table-processing software and statistics on dimers for the current query ⑧.

PFCSDB stores 14 saccharides from *S. japonicus* and *S. pombe*, and these saccharides are composed of 12 monomeric residues, α-d-mannopyranose being the most abundant. α-d-mannopyranose, α-d-galactopyranose and α-d-glucopyranose are found at the terminal positions. d-mannitol and d-2-aminoglucitol found at the reducing ends are probably analytical artifacts. If a residue is suspected to be an analytical artifact, a corresponding note is present in the full record, which can be accessed by clicking links in column ⑤.

**Figure 3.**
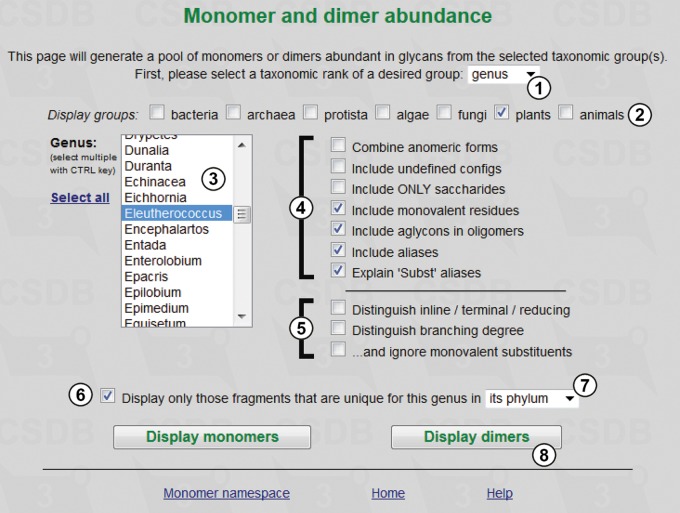
Fragment abundance form. Only fragments unique for the genus *Eleutherococcus* in its phylum will be processed.

Figure 3 exemplifies the usage of the dimer abundance tool and shows the query form for statistics on dimers found in glycans of the *Eleutherococcus* genus (①,②,③), where only fragments unique for the genus in its phylum are selected (⑥,⑦). Monovalent residues (such as methyl or acetyl groups), aglycons and other aliases are included (④); positions of residues in the structure and residue branching degree are left undistinguished (⑤). Pressing button ⑧ calculates the statistics.

**Figure 4.**
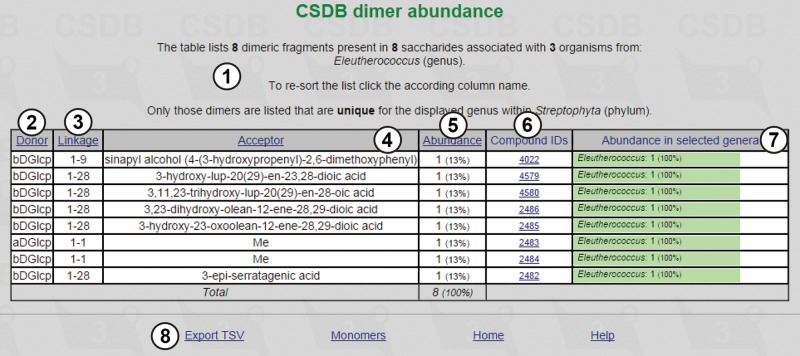
Dimeric fragments unique for the *Eleutherococcus* genus in its phylum.

The result table (Figure 4) lists dimeric fragments unique for the *Eleutherococcus* genus in its phylum (*Streptophyta*), together with statistical overview ①. It includes residues found in the structures from the organisms belonging to the specified taxon (in this case, eight dimeric fragments from eight saccharides associated with three organisms from the *Eleutherococcus* genus) and their absolute and relative (in per cent) abundance ⑤. There are compound IDs ⑥ for structures that contain the corresponding fragment (in this example, unique fragments are exotic, and each fragment belongs to one structure only), and fragment abundance among the genera ⑦ (in Figure 4, 100% for all residues, because only one genus was selected). The result table for dimer abundance includes ‘Donor’ ②, ‘Linkage’ ③ and ‘Acceptor’ ④ columns, reflecting two residues in a dimeric fragment and a linkage between them, respectively. The page also contains accessory links ⑧.

Currently, the ‘all biota’ option (drop-down list ⑦ in Figure 3) implies all biota present in the database that is being queried. Because BCSDB and PFCSDB are not merged, using this option, especially at the domain level, produces many false positives (particularly, there is almost no other biota than bacteria in BCSDB). When analysing unique fragments, you should keep in mind that ‘All biota’ includes only those domains which are populated in the current database. This option is reserved for the future; using the option ‘in its kingdom’ is preferable.

#### Coverage statistics

**Figure 5.**
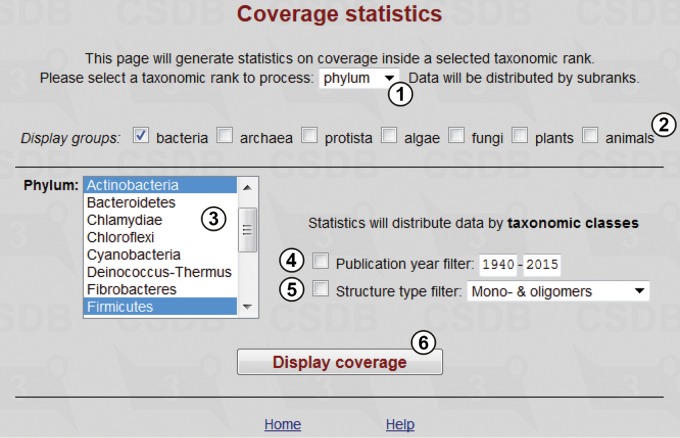
Coverage statistics form.

This tool produces statistics on coverage in various taxonomic ranks (all biota, domain, phylum, class or genus) and allows comparison of structure distribution among taxa. Figure 5 shows the query form. User can define taxonomic rank ① and then select groups ②, for which taxa ③ of this rank are displayed (in this example, two phyla, *Actinobacteria* and *Firmicutes*, are selected). The data will be distributed among the subtaxa having a lower rank than that of the selected taxa. Publication year ④ and structure type ⑤ filters allow refining the search by date of publication or structure type (the available types are mono- and oligomers, all polymers, mono- and homopolymers, cyclic polymers and biological repeats). The ‘Display coverage’ button ⑥ processes the query.

**Figure 6.**
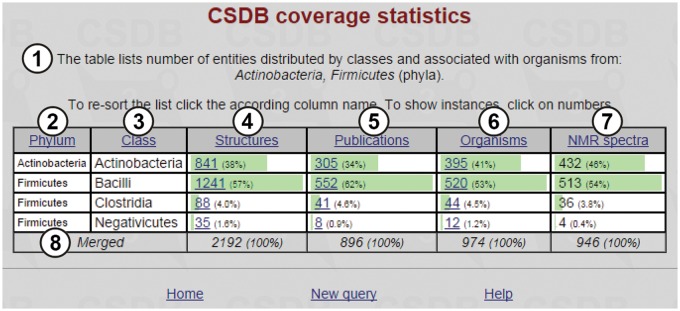
Coverage statistics for the *Actinobacteria* and *Firmicutes* phyla.

The resulting table, together with short query overview ①, is shown in Figure 6. The table includes selected taxa ② (in this case, the phyla *Actinobacteria* and *Firmicutes*; note that the data are sorted by phyla, not by default structure abundance); subtaxa ③ of the selected taxa (in this case, classes comprising the *Actinobacteria* and *Firmicutes* phyla); structures ④ (the number of structures found for a corresponding subtaxon, together with their part of the total number of structures assigned to organisms from the whole taxa selected); publications ⑤ (the number of papers in which these structures were published); organisms ⑥ (the number of taxonomically distinct organisms or groups of organisms from which these structures were obtained) and NMR spectra ⑦ (the number of NMR spectra for these structures present in the database). Clicking on the numbers leads to the corresponding database entries. Last row ⑧ shows the cumulative values.

#### Taxon clustering

This tool generates distance matrices for mono- or dimeric fragment pools from taxa populated in both BCSDB and PFCSDB databases. Basing on these matrices, selected taxa can be clustered into groups, with the corresponding dendrograms displayed. Such clustering of taxa according to their glycans can be visualized as phenetic trees and can be exported for external processing.

Briefly, the tool generates a list of taxa, in accordance with the specified constraints (Scope settings and General settings), and a list of structural fragments that will be included in the analysis, in accordance with the specified constraints (General settings and Fragment pool settings). When these two lists are prepared, the program builds mono- or disaccharide profiles (binary occurrence codes) that reflect the occurrence of particular fragments in the structures assigned to organisms belonging to every taxon under analysis. The occurrence codes are compared to obtain Hamming distances (18) between taxa; these distances are normalized by the study degree for each taxon (how many structures are assigned to it) and produce a dissimilarity matrix used for cluster analysis of taxa and for building phenetic trees.

**Figure 7.**
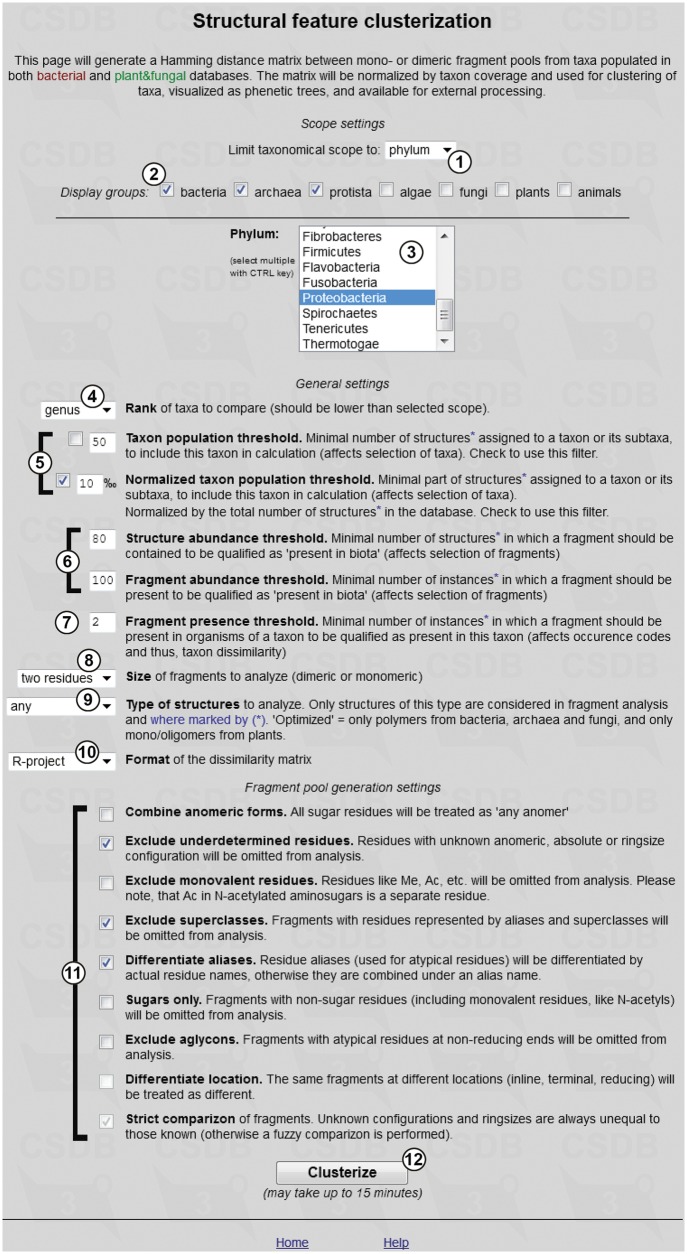
Parameter input for clustering of taxa by glycan structural features.

Figure 7 shows the input form of the tool. The scope settings define a particular taxonomic group for the analysis (by default, it is all biota, which means no limitations on taxon selection). Available taxonomical scopes ① are ‘all biota’, ‘domain’, ‘phylum’, ‘class’ or ‘genus’. If the rank ‘phylum’ or lower is selected, list ③ appears, from which individual phyla, classes or genera can be selected. Multiple selection is allowed. Checkboxes ② allow filtering these lists to the domains checked. Both databases (BCSDB and PFCSDB) are utilized.

The general settings include options and thresholds for generation of a taxon list, a fragment list and occurrence codes. The ‘Rank’ selector ④ specifies the rank of taxa being compared (kingdom, phylum, genus, species or strain). If ‘species’ is selected, the additional link ‘Specify exact species’ appears on the right. Clicking this link opens the list of all species present in CSDB in a new window, where specific species can be selected. If a species is missing from the list, currently there are no carbohydrate structures from this organism in CSDB. This option resets scope settings ①,②,③ to ‘all biota’.

Two taxon population thresholds ⑤ define how populated a taxon should be to be included in the analysis. The population is a number of structures assigned to organisms belonging to this taxon or its subtaxa. The upper field specifies the absolute minimum (the number of structures); the lower field specifies the relative minimum (the number of structures normalized by the total number of structures in the database to which the taxon belongs). Checkboxes define which threshold to use; both can be used simultaneously. Lower values produce analysis on greater variety of taxa, whereas higher values limit the taxonomical scope only to the most studied taxa. Low thresholds may lead to the data biased by non-uniform distribution of deposited structures in exotic taxa; high thresholds decrease the number of taxa under analysis, and the results even on widespread genera may be lost. Default values are pre-filled depending on the selected rank to pick up from 5 to 20 most studied taxa.

Two abundance thresholds ⑥ define how ‘popular’ a fragment should be to be qualified as present in biota and therefore to be included in the analysis. Higher abundance thresholds shift the results to widespread saccharides, avoiding analytical artifacts and atypical rarely occurring fragments. The first threshold sets the minimal number of structures in which a fragment should be present; the second threshold sets the minimal number of instances of a fragment in all structures present in CSDB. As a structure may contain several identical fragments, the second threshold should always be higher than the first one. Default values are set to cover enough fragments specific to certain taxa; these values are sufficiently high not to cover statistically unpopulated exotic fragments.

Fragment presence threshold ⑦ defines how frequently a fragment should appear in the structures belonging to a taxon to be qualified as present in this taxon (by default, it is set to 2 to avoid unapproved occurrences).

Size of fragments ⑧ specifies which fragments (monomeric or dimeric) will be used for the analysis. Monomeric fragments focus the research on the glycan composition, whereas dimeric fragments (default) focus it on biosynthesized linkages. Type of structures ⑨ defines the scopes of structures from which fragments are taken. The allowed types are ‘any’ (default), ‘only polymers’, ‘only oligomers’ or ‘optimized’. The latter implies most biologically active structures in each domain: polymers from bacteria, fungi and archaea, and oligomers from other kingdoms.

Format ⑩ specifies how the dissimilarity matrix is exported (R-project, Phylip or tab-separated values). If the default format ‘R-project’ is selected, the dendrographic visualization of results is done automatically on the result page. Phylip matrices can be processed by various software for clustering, whereas the TSV format is most universal.

Fragment pool settings 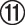 define options for generation of the fragment list. ‘Combine anomeric forms’ treats different anomers as a single residue without anomeric configuration. ‘Exclude underdetermined residues’ omits fragments containing residues with unknown anomeric (if not combined), absolute or ring size configuration from the analysis. ‘Exclude monovalent residues’ omits fragments containing (or consisting of) monovalent residues (including acetyl groups of amino sugars) from the analysis. ‘Exclude superclasses’ omits fragments containing (or consisting of) residues presented by superclasses (like HEX) or aliases (like Subst). ‘Differentiate aliases’ replaces all Subst aliases with actual alias values prior to the analysis. In this case, aliases are treated as different residues depending on their actual values; otherwise, all Subst aliases are combined under a single residue name like Subst or Subst1. ‘Sugars only’ omits fragments containing at least one non-monosaccharide residue from the analysis. Acetylated amino sugars are interpreted as dimeric fragments containing the non-sugar residue Ac and a sugar residue. ‘Exclude aglycons’ omits fragments with residues at the reducing end classified as aglycons. If unchecked, aglycons from mono- and oligomeric structures are processed together with other residues. ‘Differentiate location’ (currently disabled) processes identical fragments at different locations in the structure (inline, terminal or reducing) as different fragments. ‘Strict comparison’ (currently always enabled) implies that similarity of two fragments is evaluated by strict comparison of configurations (e.g. ?DGalp is not equal to bDGalp). When unchecked, residues with known configurations are considered a subset of those with unknown.

Pressing the ‘Clusterize’ button 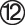 runs the analysis. The specified restrictions affect the number of taxa and fragments being processed, and the calculation may take from 30 s to 10 min.

**Figure 8.**
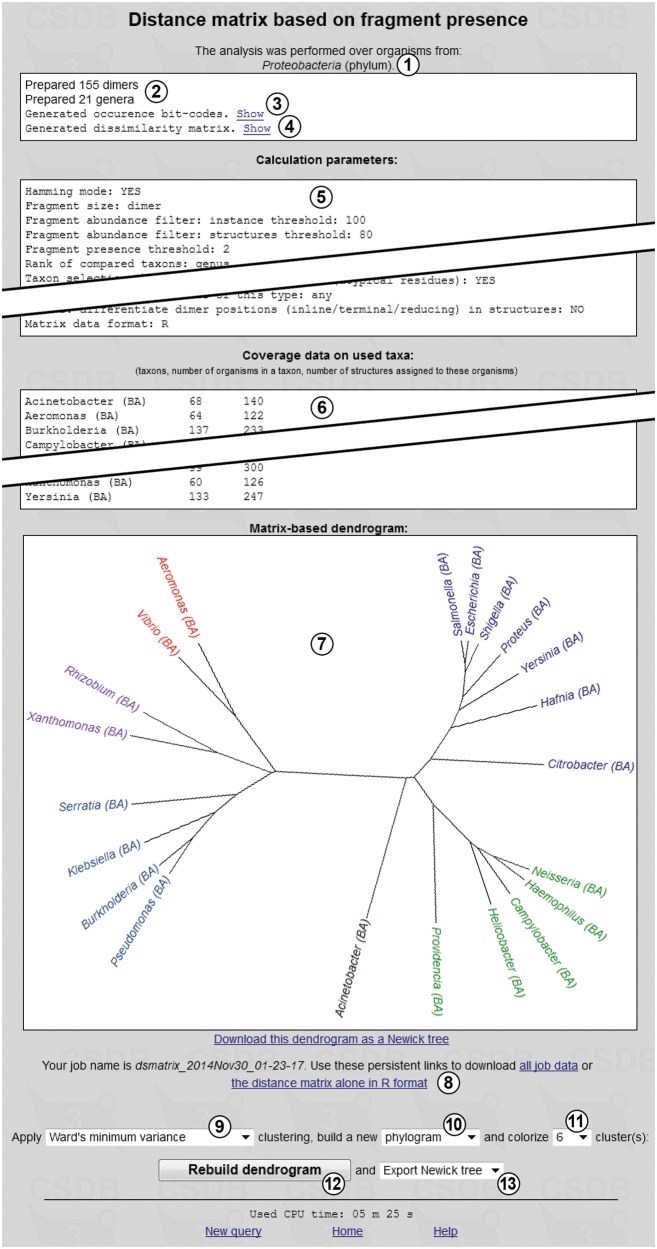
Clustering result window.

Figure 8 shows the results of the cluster analysis, the input data for which are presented in Figure 7. Overview of the taxonomic scope ① is given at the window top. Reports on the number of generated fragments and taxa ② indicate that the taxon and fragment pools were prepared without errors. Occurrence bit-code generation report ③ is accompanied by the ‘Show’ link, which displays a table with taxa and their binary glycoprofiles (occurrence codes), together with a separate list of the fragments used. Dissimilarity matrix generation report ④ is also accompanied by the ‘Show’ link, which displays the distance matrix in the selected format.

‘Calculation parameters’ ⑤ is a dump of all metadata related to the analysis, in accordance with the preferences selected in the glycome-based taxon clustering form (Figure 7). ‘Coverage data on used taxa’ ⑥ is a tab-separated table containing processed taxon names with database markers (in this case, BA, for bacterial), a number of organisms within a taxon or its subtaxa and a number of structures assigned to these organisms.

As the ‘R-project’ dissimilarity matrix format was chosen for the analysis, dendrogram ⑦ reflecting the clustering results is displayed, together with additional options ⑨–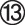. Regardless of the matrix format, the analysis results (excluding dendrograms) are stored on the CSDB server as two files referenced by links ⑧. The first file (all job data) contains a dump of the input parameters, generated dissimilarity matrix, and coverage data on taxa. The second file (in Figure 8, the distance matrix alone in the R format) contains the dissimilarity matrix, which can be later processed in other software. These files are stored for six months.

For R-formatted matrices, additional options are available. The clustering algorithm can be selected from drop-down list ⑨ [in Figure 8, Ward’s minimum variance method; default is unweighted pair group method with arithmetic mean (UPGMA), see the Experimental section for details]. Graph type selector ⑩ specifies the dendrogram type: phylogram (rectangular stems), cladogram (triangular stems), unrooted tree (as in Figure 8) or circular tree. The leaves (taxa) of the phenetic tree can be colored according to the number of clusters specified by selector 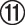 (six in Figure 8). Depending on the selected clustering method, some types of dendrograms and the coloring feature may be disabled. The ‘Rebuild dendrogram’ button 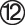 updates image ⑦ and exports the phenetic tree in the format specified by selector 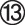 (allowed formats are ‘no export’ (default), Newick tree or Nexus tree). After the export, a link to the corresponding file appears below the image.

### Estimation of CSDB content

**Figure 9.**
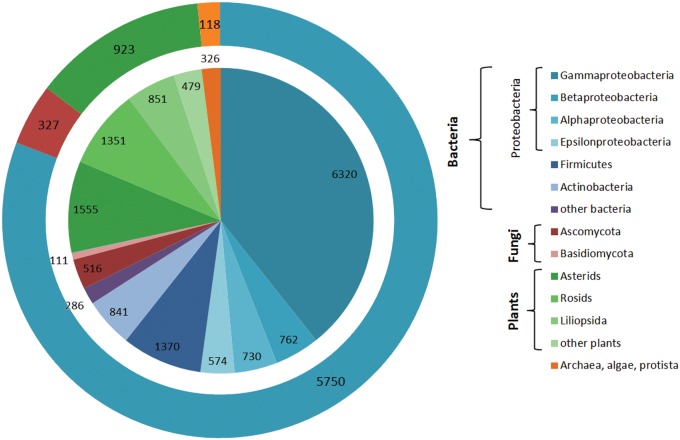
CSDB coverage by structures and organisms. Absolute numbers of structures/organisms are provided for every domain/phylum/class. The inner diagram shows distribution of structures among domains split into lower ranks; the outer ring shows distribution of organisms among domains. The following color code is used: blue shades, bacteria; red shades, fungi; green shades, plants; orange, archaea, algae and protista.

The described statistical tools were employed to estimate the CSDB coverage and content. Figure 9 shows the CSDB coverage in various domains, phyla and most populated classes. At the moment, CSDB contains ∼10 900 bacterial structures from 5750 bacteria, 4236 structures from 923 plants, 636 structures from 327 fungi and 326 structures from 118 archaeal, algal or protista species published in 6100 papers (1941–2013). BCSDB currently provides conditionally complete coverage on bacterial carbohydrate structures. The average time lag between the publication of a structure and its deposition in CSDB is one year. At the moment, PFCSDB provides complete coverage up to the year 1997 and includes the plant and fungal data exported from CarbBank (19), as well as data from selected publications up to 2009 (17).

#### Mono- and dimeric fragment distribution

The comparative analysis of mono- and disaccharide building blocks present in glycans from bacteria and mammals was reported earlier (20). We conducted a similar analysis of the bacterial carbohydrate content in comparison with plant and fungal carbohydrates. The ‘Fragment abundance’ tool was used for assessing the monomeric and dimeric composition of all carbohydrate structures deposited in CSDB, according to taxonomic domains (bacteria, archaea, protista, fungi and plants+algae). The abundance of monomers and dimers was normalized to the total number of structures from organisms belonging to the corresponding domain, thus giving the fragment frequency. The monovalent residues, such as methanol or acetic acid, were excluded from calculation due to their high frequency, non-carbohydrate nature and domain-unspecific distribution.

**Figure 10.**
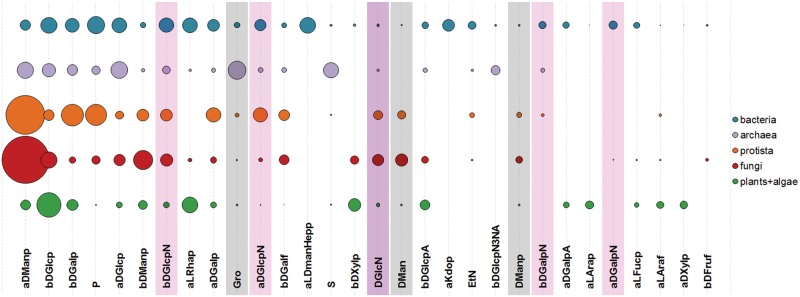
Thirty most widespread monomeric residues in carbohydrate structures from major taxonomic groups. Bubble area corresponds to averaged monomer frequency in the domain (see text), varying from 9.7 (aDManp, α-D-mannopyranose) to 0.12 (bDFruf, β-D-fructofuranose). Amino sugar residues include both acetylated and non-acetylated forms and are highlighted in lilac; undetermined residues with unknown anomeric, absolute or ring size configuration are shaded.

Figure 10 shows 30 most widespread monomeric residues typical for carbohydrate structures from organisms belonging to the six domains present in BCSDB and PFCSDB. The distribution of monomers supports the known fact that bacterial glycans are most diverse in their monomeric composition (20). Such diversity allows bacteria to occupy numerous different niches and to survive under the selection pressure induced by the host immune system and competition (21, 22). In bacterial glycans, the most widespread monomeric residues include the lipid A alias, l-glycero-d-*manno*-heptose and 3-deoxy-d-*manno*-oct-2-ulosonic acid (Kdo) not found in other domains. All these monomers are present in the LPS of Gram-negative bacteria (20, 23). In Figure 10, Lipid A was removed from consideration because it is an ambiguous compound and includes both sugar and lipid parts.

**Figure 11.**
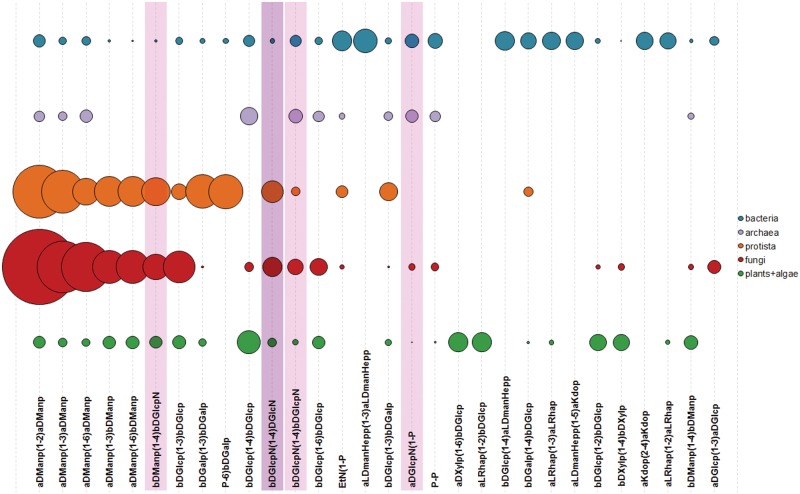
Thirty most widespread dimers in carbohydrate structures from major taxonomic groups. Bubble area corresponds to disaccharide frequency in the domain (see text), varying from 3.15 (aDManp(1-2)aDManp) to 0.09 (aDGlcp(1-3)aDGlcp). Dimers containing amino sugars, which include both acetylated and non-acetylated forms, are highlighted in lilac; dimers containing undetermined residues with unknown anomeric or ring size configuration are shaded.

Figure 11 shows 30 most widespread dimers from carbohydrate structures present in CSDB. Dimeric fragments unique for a taxonomic group can reflect peculiarities of interactions of its organisms with the environment realized by activities of group-specific GTs. At the level of domains, top 10 dimers not present in glycans from any other domain are given in Table 1. Although algae are interpreted as a separate domain in CSDB, currently there are no disaccharides unique for this domain due to a limited number of algal structures deposited in the database. Dimers containing alditols at the reducing end were omitted from the statistics because they mostly represent analytical artifacts.

**Table 1.** Top 10 unique dimeric fragments found in each domains of life present in CSDB, in abundance descending order

Bacteria	aLDmanHepp(1-3)aLDmanHepp, bDGlcp(1-4)aLDmanHepp, aLDmanHepp(1-5)aKdop, aKdop(2-4)aKdop, aLDmanHepp(1-7)aLDmanHepp, bDGlcpN(1-6)aDGlcpN, P-6)aLDmanHepp, bDGlcpN(1-3)bDGalp, aKdop(2-6)bDGlcpN, P-4)bDGlcpN
Archaea	S-6)aDManp, S-2)aDManp, bDManpNA(1-4)bDGlcpN3NA, bDGlcpN3NA(1-3)bDGlcpN, bDGlcpN3NA(1-4)aDGlcpN3NA, bDGlcpN3NA(1-3)bDGalpN, P-1)dolichol C55-60, bDS6Fucp(1-4)bDGlcp, aDDgalHepp(1-2)bDS6Fucp, aDManp(1-3)bDS6Fucp
Protista	aDGlcpN(1-6)phosphatidylinositol, bDXylp(1-2)aDManp, bDXylp(1-4)aDManp, Aep(1-P, aDArap(1-2)bDGalp, aDGlcpA(1-2)bDGalf, P-1)Dce, bDArap(1-2)bDGalp, bDManp(1-2)DManp, aDGalf(1-2)aDManp
Fungi	bDXylp(1-2)aDManp, bDXylp(1-4)aDManp, aDGlcpA(1-2)bDGalf, bDGalf(1-2)aDManp, aDGalf(1-2)aDManp, bDXylp(1-6)aDManp, bDXylp(1-3)aDManp, aDGalf(1-6)aDManp, aDManp(1-1)2-(4-trifluoroacetamido-phenyl)ethanol, aDManp(1-2)bDXylp
Plants	aDXylp(1-6)bDGlcp, aLRhap(1-2)bDGlcp, aLRhap(1-2)aLArap, bDGalp(1-2)aDXylp, bDXylp(1-2)bDManp, bDGlcp(1-3)diosgenin, bDGlcp(1-3)aLArap, aLRhap(1-2)bDFucp, Me(1-3)bDDigp, aLAraf(1-2)bDXylp

**Figure 12.**
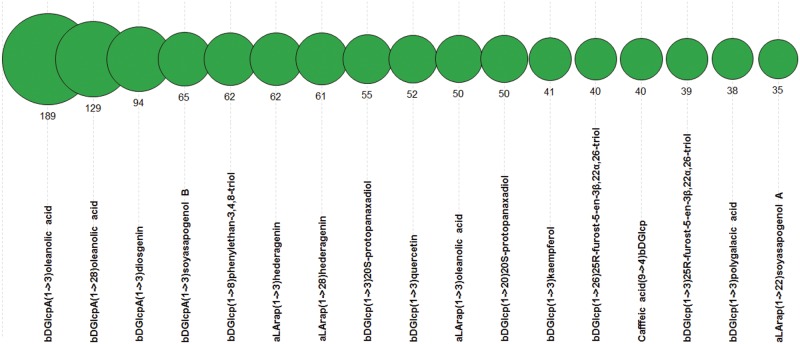
Seventeen most widespread dimeric fragments containing non-sugar moieties in plant saccharides. Numbers are absolute abundance in plant structures.

Plants are the known source of glycosides containing various aglycons of triterpene, steroid, flavonoid or phenolic origin and possessing anti-cancer (24–26) and other types of bioactivity. Therefore, we also analysed the PFCSDB coverage on plant aglycons. Figure 12 shows 17 most widespread dimeric fragments with non-sugar moieties found in plant saccharides from PFCSDB. Dimers with amino acids at the reducing end were omitted from the analysis because in most cases they present fragments from protein glycosylation sites. According to the statistics, three monosaccharide residues, β-d-glucopyranosyluronic acid, β-d-glucopyranose and α-l-arabinopyranose, form linkages with non-sugar aglycons in plant glycosides most often.

More data rows for Figures 10–12 are available as tables in the Supplementary Materials.

#### Carbohydrate-based phylogenetics

Though as of 2014 the CAZy database contains the data on almost 340 000 CAZy, only ∼4% of them are characterized biochemically, and the gap between the number of putative CAZy sequences and enzymes with established structure and functions continues to grow (13). For example, the CAZy database includes almost 1800 GTs assigned to *Helicobacter pylori*, but only 17 of them are characterized biochemically (see Supplementary Table S1). Supplementary Table S2 contains disaccharide fragments from carbohydrates found in *H. pylori*, together with possible GTs synthesizing these disaccharide fragments. It is evident that though the data on resolved carbohydrate structures and predicted GTs mostly support each other, there are disaccharides for which no enzyme has yet been predicted (e.g. mannosyl-, rhamnosyl- or ribosyltransferases), as well as predicted enzymes for which no product has yet been detected (e.g. β-1,4-n-acetylgalactosaminyltransferases). Therefore, the decryption of the glycome code is far from completion; however, bringing the enzymatic and carbohydrate ends together could promote the process. Two organisms possessing similar saccharides should possess enzymes with similar activities, and therefore studying the carbohydrate-based alliance between taxa may facilitate the investigation of carbohydrate processing proteins with yet unknown functions. For instance, *Neisseria gonorrhoeae* and *Neisseria meningitides*, two genetically close species, possess a similar set of disaccharides derived from their carbohydrate structures, and have GTs with similar predicted activities, whereas *Staphylococcus aureus* differs from the *Neisseria* species both in disaccharides and predicted activities of GTs (Supplementary Table S3). These considerations urged us to develop the ‘Taxon clustering’ tool on the CSDB platform. In 93% of structures present in CSDB, all glycosidic linkages are fully determined (regarding monosaccharides, their three configurations and linkage positions) with the exception of anomeric and ring size configurations of mutarotating residues at reducing ends. This coverage allows statistically credible calculations based on disaccharide fragments.

**Figure 13.**
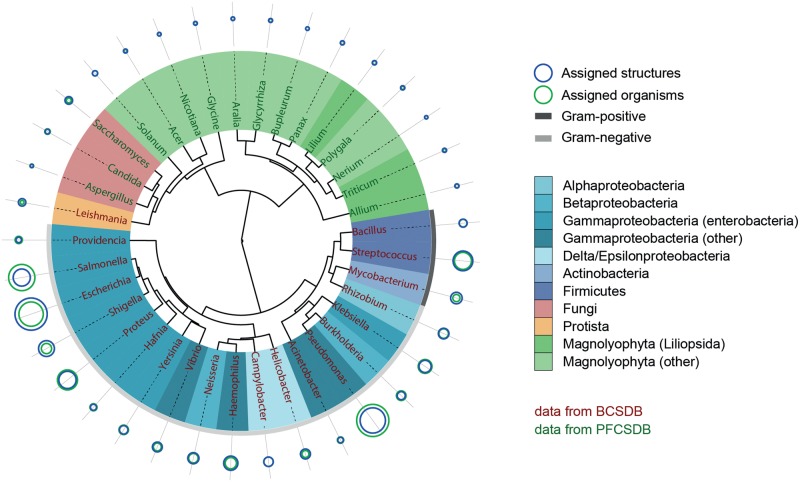
Glycome-based clustering of most studied genera. Shades of blue represent various bacterial taxonomic groups, shades of green represent plant groups, red and orange represent fungi and protista domains, respectively. The outer arc for bacteria is colored according to the Gram-reaction. Size of the circles reflects the normalized popularity of a given genus in CSDB in terms of assigned organisms (green) and assigned structures (blue). When a blue circle is not visible, it is the same size as a green one. Color of labels denotes the database from which the data came.

We selected dimeric fragments for characterization of glycome, because the monomeric composition does not reflect the information on GTs, whereas the composition of fragments larger than dimers does not add valuable data to that obtained from the dimeric composition. Clustering of most populated genera using the similarities in carbohydrate-containing dimers resulted in a phenetic tree composed of three big clusters: bacteria, plants and mixed taxa from plants, fungi and protista (Figure 13).

When gathering this statistics, we used the following filters to cover 37 most studied genera and 118 most widespread fragments:

- taxon rank = *genus*;
- taxonomic scope = *all biota* (no restrictions applied);
- minimal number of carbohydrate structures assigned to organisms belonging to a genus = 1.2% of total structures in the corresponding database (∼130 structures for the bacterial database and ∼65 structures for the plant and fungal database);
- structure type = *any *(no restrictions on the molecule part: polymeric or oligomeric);
- population of a fragment in the database to consider it statistically present in biota = at least 120 fragment instances in at least 100 structures;
- fragment size = *two residues* (dimeric fragments);
- comprising residue filters: anomeric forms were not combined, residues with missing configurations were excluded, monovalent residues were included, superclasses and aliases were excluded, aglycons were included;
- minimal occurrence of a fragment in a taxon to be qualified as present = 2 instances.

The Ward’s minimum variance method (27) was used for clustering. Lengths of branches connected to leaves were ignored for visual clarity. The details on building the dissimilarity matrix and phenetic tree are provided further in the text (see discussion of bacterial species clustering).

Most of the clades roughly matched the taxonomic lineages of organisms. Particularly, most enterobacteria appeared in a single cluster, and the genera most genetically or antigenically close to each other (*Escherichia, Shigella* and *Salmonella*) (28–31) occupied a small clade with short branch lengths. Although *Burkholderia* and *Pseudomonas* belong to different classes, they tend to co-colonize hosts (32), and many strains of the former were reclassified to the latter based on genotyping (33–35). This may explain the resemblance of glycomes of these genera as deposited in the database and their clustering in a small clade. The co-clustered *Bacillus* and *Streptococcus* are the only strictly Gram-positive bacterial genera among those analysed (*Mycobacteria* do not display the empirical Gram-reaction and are classified as Gram-positive due to the absence of the outer membrane). Phylogenetic relationship between *Leishmania* and fungal genera may be explained by shared structural features in glycolipids from organisms of these taxonomic groups (36). It is not possible to draw any reasonable conclusions from the carbohydrate-based grouping of fungal and plant saccharide structures because the CSDB coverage on the organisms from these domains is incomplete. Thus, at the moment we can only make preliminary observations, e.g. that the fungal genera *Aspergillus*, *Candida* and *Saccharomyces* are clustered together with the plant genera *Solanum*, *Acer*, *Nicotiana* and *Glycine*, since, according to the CSDB content, their carbohydrate structures are rich in mannodisaccharides.

Due to heterogeneity of species within genera and to look more thoroughly into the possibilities of grouping taxa by their carbohydrate content, we applied clustering at the level of species and then compared it to a canonical small ribosomal subunit RNA-based phylogenetic tree, following an example of Aguilar and colleagues who analysed phenetic trees based on metabolic properties for several organisms from three domains (37).

For this purpose, we used data from the bacterial part of BCSDB due to its completeness to avoid an annotation-induced bias. Thirty-three bacterial species were selected by their population in the database (at least 20 structures assigned to a taxon or its subtaxa). Some bacterial species are studied significantly more thoroughly than others; therefore, their structures in the database contain more rarely occurring fragments. This leads to longer distances between these and other species in dissimilarity matrices. To avoid such population-induced bias, we normalized the distances by the number of structures assigned to the organisms belonging to two species being compared. Another factor diminishing this bias is the fragment presence threshold, which determines how populated a fragment should be within a taxon to be included in its glycoprofile and to produce a positive mark in the occurrence code. Setting this threshold higher than 1 reduces the effect of analytical artifacts and atypical fragments.

**Figure 14.**
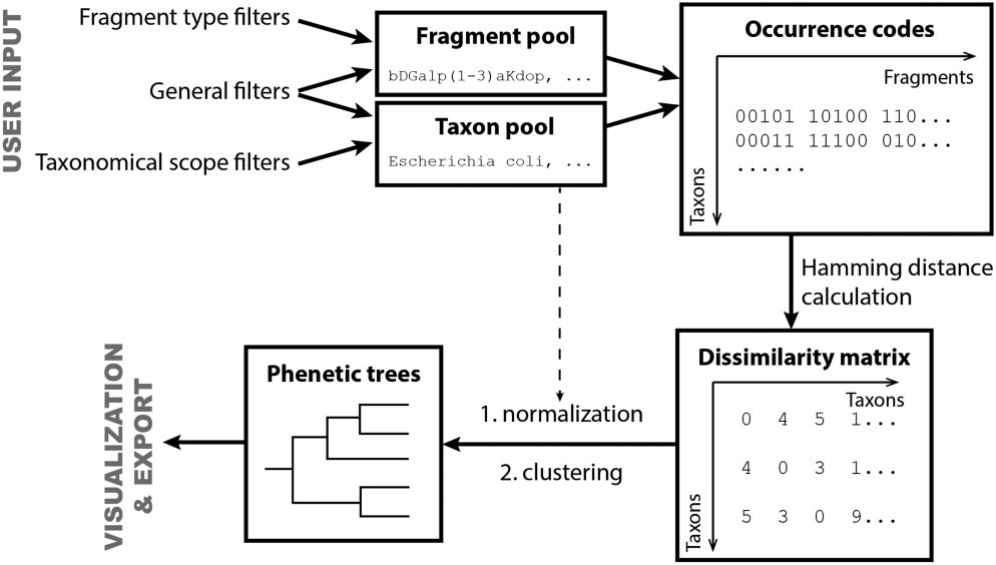
Flow chart of data processing in the reported online tool.

We prepared matrices, which reflect the dissimilarity of taxa, by using the reported online tool on the content of CSDB. The underlying algorithm is summarized in Figure 14. At first, the fragment and taxon pools were prepared according to the user input. The filters and thresholds that affect population of these pools are described in the ‘Taxon clustering’ section.

To select taxa, we specified the explicit list of species. This list was obtained by manual curation of the available ribosomal RNA (rRNA) data (see below) for the species pre-selected with the following parameters:

- taxon rank = *species*;
- taxonomic scope = *all biota* (no restrictions applied);
- minimal number of carbohydrate structures assigned to organisms belonging to a species = 20.

To select fragments, we used the following filters:

- structure type = *any *(no limitation on the molecule part: polymeric or oligomeric);
- population of a fragment in the database to consider it statistically present in biota = at least 120 fragment instances in at least 100 structures; lowering these thresholds leads to inclusion of non-representative fragments and analytical artifacts, while making them higher leads to loss of taxon-specific fragments, which are the clustering basis;
- fragment size = *two residues* (dimeric fragments);
- comprising residue filters: anomeric forms were not combined, residues with missing configurations were excluded, monovalent residues were included, aglycons and aliases were included and differentiated.

118 fragments from the fragment pool were checked for presence in the structures assigned to organisms belonging to 33 taxa from the taxon pool. The fragment was considered present in a taxon if its abundance in structures from this taxon was equal to or exceeded 2 (filter ‘Fragment presence threshold’). The fragment presence was encoded as a binary string (occurrence code) specific for every taxon. These disaccharide composition phylogenetic profiles were obtained similarly to protein function phylogenetic profiles reported by Pellegrini *et al.* (38): every fragment matched a certain bit position in the string; for present fragments, the corresponding bits were set to 1, for absent fragments—cleared to 0.

The occurrence codes were treated similarly to the work by Aguilar and colleagues (37). The taxa were compared pairwise using the Hamming distance between the fragment occurrence codes to give a symmetrical distance matrix. The Hamming distance between two bit strings is a number of bit inversions required to convert one string to the other (18); it is a standard method to evaluate dissimilarity.

Every cell in the dissimilarity matrix was normalized by the total number of structures assigned to both taxa matching this cell to avoid the study-induced bias (see above), and the whole matrix was normalized to a maximal distance 100 for compatibility with other clustering software.

**Figure 15.**
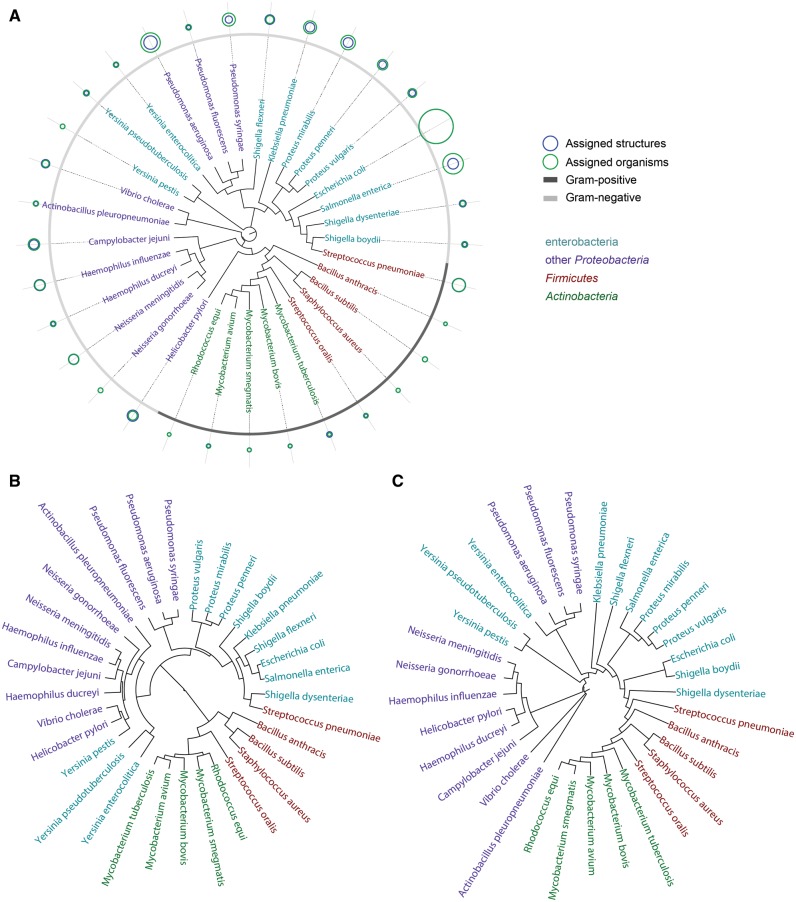
Circular phenetic trees based on dimers (containing monosaccharides, aliases, aglycons, monovalent residues) present in compounds from bacterial species most populated in BCSDB. *Firmicutes* are shown in red, *Actinobacteria* in green, *Enterobacteria* in cyan and other *Proteobacteria* in violet. Three dendrograms correspond to different clustering methods: NJ (**A**), Ward’s minimal variance (**B**) and balanced minimum evolution (**C**). Blue and green circles in (A) depict the normalized number of structures and organisms assigned to each species, correspondingly. When a blue circle is invisible, it is the same size as a green one. Gray color code in the outer rim (A) reflects the Gram-reaction. The underlying numbers and trees are available in Supplementary materials.

The normalized dissimilarity matrix reflecting the disaccharide composition of taxa was imported into the R environment (39) to be analysed by most common clustering algorithms for distance data. We built carbohydrate-based phenetic trees from the distance matrix by seven clustering algorithms: hierarchical agglomerative complete linkage clustering; UPGMA (40); Ward’s minimum variance method (27); classical (41) and improved [biological neighbor joining (BIONJ)] (42) neighbor joining (NJ); ordinary least-squares and balanced minimum evolution (43). Other clustering algorithms were not applied due to their poor applicability to biological phylogenetics (agglomerative hierarchical clustering with other types of linkage interpretation) or dedication to handling incomplete distance matrices (minimum variance reduction) (44, 45). Figure 15 represents circular phenetic trees obtained by three representative clustering methods, together with cumulative data on the BCSDB coverage related to the examined species (Figure15A).

The default method for the web interface is hierarchical average linkage clustering (UPGMA) (40). This algorithm analyses the structure of a pairwise dissimilarity matrix and constructs a rooted ultrametric tree. At each step, the two nearest clusters are combined into a higher-level cluster, based on the average distance between elements of each cluster. UPGMA was reported to produce adequate results for genome-based clustering of bacteria on the subspecies level (46). The main disadvantage of UPGMA is the ‘molecular clock’ assumption, which pre-supposes a constant rate of changes in all lineages. This assumption may be useful for genetic studies (47, 48), but not for phenetic trees based on differences in carbohydrate structures the occurrence rates of which are unlikely to be constant.

Alternative approach to hierarchical clustering is the Ward’s method, in which the choice of clusters to be merged is based on the least possible increase in the overall within-cluster variance. This method is particularly efficient if the data set includes no major outliers (49). In 2014, the Ward’s minimum variance method was reported in regard to functional classification of phytoplankton species (50).

The NJ algorithm is free from ‘molecular clock’ restrictions and is widely accepted as a standard method for distance data clustering in biology (41). It is a bottom-up clustering method, which is usually used to construct trees based on DNA sequence data. Its improved version, BIONJ, chooses the reduction, which minimizes the variance of a new distance matrix at each step of matrix reduction, allowing better estimations for selection of the pair of taxa to be agglomerated during the next steps (42). The ability of both UPGMA and NJ methods to differentiate various taxa depends on the underlying structure of the data used to construct the dissimilarity matrix (46, 51).

The main disadvantage of the NJ methods is their ability to produce branches with negative lengths which leads to occasional failure to find an optimal tree. A more recent minimum evolution method (43) was employed to overcome these drawbacks. This method is based on the assumption that the correct tree is the one that exhibits the minimal total amount of evolution. However, it has to be mentioned that it still produced negative branch lengths in some of our examples. In the balanced weighting minimum evolution scheme, sibling subtrees have equal weights, as opposed to the standard ‘unweighted’ ordinary least squares scheme, where all taxa have the same weight so that the weight of a subtree is equal to the number of its taxa.

Overall, in our experience the methods produce similar, but not identical trees, which differ in relative positioning of more distant clusters, while consistently identify the same pairs of taxa as the most closely related (Figure 15).

**Figure 16.**
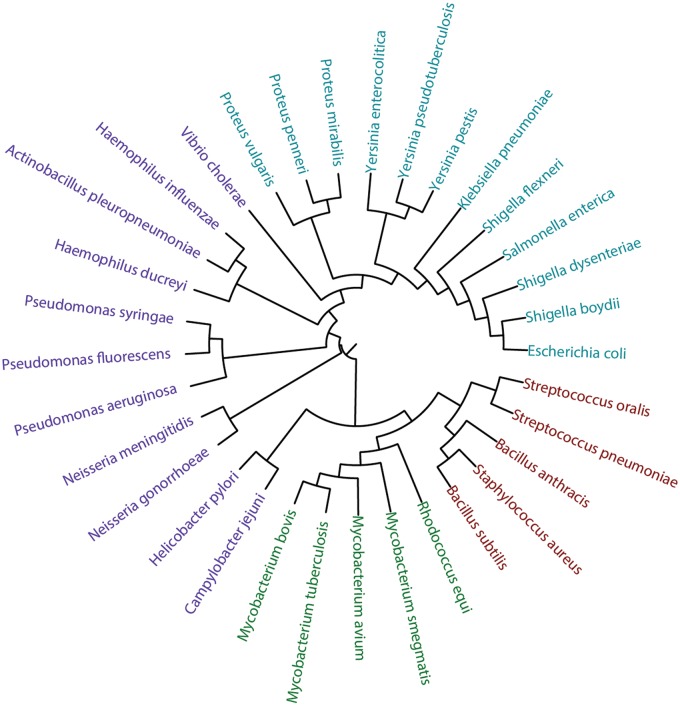
Phylogenetic tree based on small ribosomal subunit rRNA sequences. *Firmicutes* are shown in red, *Actinobacteria* in green, *Enterobacteria* in cyan and other *Proteobacteria* in violet. The underlying data are available in the Supplementary materials.

We compared the trees obtained by each method to a canonical phylogenetic tree based on small ribosomal subunit rRNA homology for the selected 33 bacterial species (Figure 16; for SILVA numbers of the rRNA sequences and sequences themselves in the FASTA format, see the Supplementary materials). The mean topological score between internal branches under the one-to-one mapping of the trees was used as a similarity metrics. It was produced by pairwise mapping and topological scoring of branches as described (52). We did not apply sophisticated comparison methods, because this article presents tools for gathering and visualization of statistical data, while their interpretation requires special biochemical and microbiological experience and is beyond its scope. The similarity between the RNA-based tree and carbohydrate-based trees clustered by various methods was 53% (complete linkage), 52% (UPGMA), 57% (Ward), 59% (NJ), 51% (BIONJ), 44% (balanced minimum evolution) and 46% [ordinary least squares (OLS) minimum evolution]. Filtering the fragment pool to purely carbohydrate fragments (disaccharides) introduced only minor changes to these values.

It can be seen, that the carbohydrate trees partially agree with the rRNA one. The phyla *Firmicutes* and *Actinobacteria* are clustered together, with the exception of *Streptococcus pneumonia*, whose carbohydrate content resembles that of *Shigella*. Both rRNA and carbohydrate sequences converge *Escherichia coli* to *Shigella*, whereas species from *Pseudomonas*, as well as from *Proteus*, are close to each other both by rRNA and glycans. The phylogeny of species constructed by using the Ward’s criterion is most close to that of genera and to existing knowledge on bacterial taxa localization in the tree of life.

The similarity between the two types of the tree of life is expected as the repertoire of carbohydrate structures in an organism is defined by its CAZy. It was demonstrated that genome-based and rRNA-based phylogenetic trees correlate well with each other (37, 53), and differences in CAZy genes reflect the differences in the whole genomes—but only to some degree. During evolution, the selection pressure differently affects different genes and therefore their products, and two organisms that differ significantly in the genome content may possess similar phenotypic features (54, 55).

Thus, the difference observed between the carbohydrate-based and rRNA-based trees may reflect the fact that carbohydrates are the main bacterial instrument for interacting with the environment (11), and expression of certain carbohydrate structures may correspond to certain habitat/activities. For example, *Neisseria gonorrheae* and *Haemophilus ducreyi*, which are not very close in the rRNA-based tree of life but evidently possess similar glycans, both inhabit the urogenital track of humans where they cause sexually transmitted diseases (56, 57) and therefore meet similar challenges. Because the exact mechanisms of bacterial pathogenesis in most cases are not understood at the molecular level, such proximity of species in the carbohydrate phenetic tree suggests the similarity of the ways of their action and allows proposing directions for future experimental study. Thus, the carbohydrate-based phenetic trees may demonstrate similarity and differences in both pathogenic and CAZy activities.

## Conclusions

In this article, we describe three new CSDB instruments: ‘Fragment abundance’, ‘Coverage statistics’ and ‘Taxon clustering’. These tools are dedicated to statistical analysis of the carbohydrate content in different taxonomic groups, as well as to studying carbohydrate-based relationships between bacteria, fungi and plants. We suppose this information may be useful for eliminating the gap between the accumulated structural and biochemical data on carbohydrate-processing enzymes. We do not draw any far-reaching conclusions from the obtained carbohydrate data, because it requires a thorough analysis by specialists in the field of bacterial pathogenesis. Still, we have no doubts that in the future these instruments would be fruitfully used by glycoscientists and bacteriologists.

## Experimental

### Programming

Presented tools were implemented as web-services on the CSDB platform using the PHP 5.4 programming language, MySQL 5.5 database engine and DHTML/Javascript for web-pages. The tools were tested in modern versions of Mozilla Firefox, Google Chrome and Microsoft Internet Explorer. The online help is available in the ‘Statistical tools’ section of the CSDB help: http://csdb.glycoscience.ru/bacterial/core/help.php?topic=stat

### Estimation of CSDB coverage

Data were exported as tab-separated values from the web-interface of the presented tools, were joined for BCSDB and PFCSDB using a dedicated script in PHP 5.4 and were processed and visualized in Microsoft Excel 2010.

### rRNA phylogenetic tree

Small subunit rRNA sequences were obtained from the SILVA database (http://www.arb-silva.de/; release 119) (58). The corresponding accession numbers are listed in the Supplementary materials. Phylogeny analysis was performed at Phylogeny.fr (http://phylogeny.lirmm.fr/) (59) in the ‘One-Click’ mode with default options, implying BIONJ clustering method; the resulting tree was exported in the Newick format and processed with the iTOL online software (60).

### Clustering and building dendrograms

Dissimilarity matrices were imported into the R environment (39) and analysed by multiple clustering algorithms. The hierarchical clustering was implemented via the built-in *hclust* function in R [using UPGMA (40), complete linkage (40), or ward.D2 methods (27)]. The NJ and minimum evolution methods utilized the BIONJ (42) and balanced fastME (43) functions, correspondingly, as implemented in the Analyses of Phylogenetics and Evolution (*ape*) library (61) for R. The data were visualized online and were exported using built-in functionality provided in *ape*. Tree comparison was carried out using the approach of Nye and coworkers (52) as implemented in the Compare2Trees tool (http://www.mas.ncl.ac.uk/∼ntmwn/compare2trees/index.html) after conversion to the Newick format. Complex dendrograms were built using the iTOL service (60, 62) on distance matrices exported online in the Nexus format and other supplementary data generated online. The trees were presented as rooted to avoid label overlap; the terminal branch lengths were ignored on plots for visual clarity. Unrooted trees and trees with variable terminal branch lengths can be easily restored from the source data (see the Supplementary Materials).

## Abbreviations

Residue names
Aep, 2-amino-ethylphosphonic acid
aDArap, α-D-arabinopyranose
aDDgalHepp, D-glycero-α-D-galacto-heptopyranose
aDGalf / bDGalf, D-galactofuranose (α- and β-anomers)
aDGalp / bDGalp, D-galactopyranose (α- and β-anomers)
aDGlcp / bDGlcp, D-glucopyranose (α- and β-anomers)
aDGlcpA / bDGlcpA, D-glucopyranosyluronic acid (α- and β-anomers)
aDGlcpN / bDGlcpN, 2-deoxy-2-amino-D-glucopyranose (α- and β-anomers, alone or within N-acetylated fragment)
aDGlcpN3NA / bDGlcpN3NA, 2,3-dideoxy-2,3-diamino-D-glucopyranosuronic acid (α- and β-anomers)
aDManp / bDManp, D-mannopyranose (α- and β-anomers)
aDXylp / bDXylp, D-xylopyranose (α- and β-anomers)
aKdop, 3-deoxy-α-D-mannopyranosyl-oct-2-ulosonic acid
aLArap, α-L-arabinopyranose
aLAraf, α-L-arabinofuranose
aLDmanHepp, L-glycero-α-D-manno-heptopyranose
aLFucp, 6-deoxy-α-L-galactopyranose
aLRhap, 6-deoxy-α-L-mannopyranose
bDDigp, 2,6-dideoxy-β-D-ribo-hexopyranose
bDFruf, β-D-fructofuranose
bDFucp, 6-deoxy-β-D-galactopyranose
bDManpNA, 2-deoxy-2-amino-β-D-mannopyranosuronic acid
bDS6Fucp, 6-deoxy-6-sulpho-β-D-galactopyranose
Dce, cis-decenoic acid
DGlcN, D-glucosamine with unknown anomeric and ring size configurations
DMan, D-mannose with unknown anomeric and ring size configurations
DManp, D-mannopyranose with unknown anomeric configuration
EtN, 2-aminoethanol
Gro, glycerol with unknown absolute configuration
P, phosphoric acid
S, sulfuric acid

## Funding

This work and open-access publication was supported by Russian Science Foundation (RSF grant 14-50-00126).

## Supplementary Data

Supplementary data are available at *Database* Online.

*Conflict of interest*. None declared.

Supplementary Data
