# Supplementary material for: Carbohydrate Structure Database: tools for statistical analysis of bacterial, plant and fungal glycomes
Source: Database (Oxford). 2015 Sep 3;2015:bav073. doi: 10.1093/database/bav073 (PMC4559136; doi:10.1093/database/bav073)
Supplement: Supplementary Data [file supp_2015_bav073_index.html]

Carbohydrate Structure Database: tools for statistical analysis of bacterial, plant and fungal glycomes — Supplementary Data 

# Carbohydrate Structure Database: tools for statistical analysis of bacterial, plant and fungal glycomes

## Supplementary Data

files

- Supplementary Data - zip file
