# Supplementary material for: Carbohydrate Structure Database: tools for statistical analysis of bacterial, plant and fungal glycomes
Source: Database (Oxford). 2015 Sep 3;2015:bav073. doi: 10.1093/database/bav073 (PMC4559136; doi:10.1093/database/bav073)
Supplement: Supplementary Data [file supp_bav073_all_supplementary_data.zip › all_supplementary_data/GT_tables.rtf]

Table S1. Statistic data on glycosyltransferases found and predicted for Helicobacter pylori*
GT family	GT number	Of them characterized	Comment	
GT2	67	0		
GT4	68	3	Includes cholesterol áglucosyltransferases	
GT8	394	3	LPS biosynthesis proteins	
GT9	302	1		
GT10	136	6		
GT11	68	2		
GT19	68	1	Possible lipid A disaccharide synthases	
GT25	320	1		
GT28	68	0	Includes possible undecaprenyldiphospho-muramoylpentapeptide âNacetylglucosaminyl transferases	
GT30	68	0	Include possible 3deoxy-Dmanno-octulosonic-acid transferases	
GT42	13	0		
GT51	101	0	Include possible penicillin-binding proteins	
GT82	41	0	Include possible â1,4Nacetylgalactosaminyl transferases	
GT_NC	63	0		
* Based on information obtained from the CAZy database in March 2015.


Table S2. Disaccharides and corresponding characterized and predicted glycosyltransferases found in Helicobacter pylori*
Donor	Linkage	Acceptor	Glycosyltranferases (GenBank/UniProt)**	
aDDmanHepp	1-2	aDDmanHepp	GT9: AAD07549.1/O25224, AAX12679.1/Q5D6B0, ADZ50261.1, ADZ49368.1, ADZ51869.1, ADZ50969.1, AAD07342.1/O25056, AAD08237.1/O25802, AJF09417.1, AJF09505.1, AJF10958.1, AJF11046.1, ADU41556.1, ADU40654.1, ADU41461.1, ACX97491.1/D0IRE8, ACX98281.1/D0ITN8, ACX98900.1/D0JYI0, ACX99679.1/D0K0Q9, ACX99065.1/D0JYZ5, AEE70903.1, AEE70807.1, AEE69937.1, ADN79423.1/E1S7E3, ADN80404.1/E1S614, ADN80317.1/E1S9Y7, AFX91513.1, AFX90693.1, AFX89301.1, CAX28829.1/C7BYL9, CAX29815.1/C7C096, CBI65857.1/D7FCF0, CBI66840.1/D7FF83, AHA87976.1, AHA87527.1, AHA87687.1, AHZ27755.1, AHZ27907.1, AHA89259.1, AHA89548.1, AHA89098.1, AHZ27290.1, ADO04360.1/E1Q852, ADO03520.1/E1Q9P4, AFF20600.1, AFF20758.1, BAJ55726.1, BAJ54883.1, BAJ56298.1, BAJ57114.1, BAJ57870.1, BAJ58706.1, BAJ60228.1, BAJ59406.1, ACI27025.1/B5ZA45, ACI27886.1/B5Z8I7, ACI27201.1/B5Z6K2, ADU82017.1, ADU81160.1, AJD66044.1, AJD66128.1, ABF84348.1/Q1CUM4, ABF85199.1/Q1CS73, ABF84523.1/Q1CU49, AFI06987.1, AFI07806.1, ADU79509.1, ADU80449.1, AHZ25937.1, AHZ25850.1, AAD05843.1/Q9ZMF6, AAD06697.1/Q9ZK26, AAB65778.1/O30355, ADU82729.1, ADU83580.1, BAO97204.1, BAO97917.1, BAO97297.1, ADU82904.1, AAX12681.1/Q5D6A8, BAM96196.1, BAM97057.1, BAM98515.1, BAM97711.1, AHN35146.1, AHN36622.1, AHN37987.1, AHN39429.1, AHN40921.1, AHN42327.1, AHN43769.1, AHN45294.1, ACJ08309.1/B6JN31, ACJ07436.1/B6JKK8, AFI02069.1, AFI02915.1, ADO07374.1/E1Q3C6, ADO06530.1/E1Q539, AAX12680.1/Q5D6A9, AEN14924.1, AEN15757.1, AEN17991.1, AEN18885.1, AFV43096.1, AFV44002.1, AFV44690.1, AFV45595.1, ADO05028.1/E1PUI1, ADO05860.1/E1PWZ5, AFI01376.1, AFI00560.1, AFH99016.1, AFH99846.1, AFH98291.1, AFH97437.1, ACD48635.1/B2UUV3, ACD47742.1/B2USB0, ADO01898.1/E1PXZ9, ADO02755.1/E1Q0R4, AGL70824.1, AGL70916.1, AEN16487.1, AEN17320.1, AGT73551.1, AGT74495.1, ADU85141.1, ADU84307.1, AGL66809.1, AGL66716.1, AGL69452.1, AGL69358.1, AGL65939.1, AGR62916.1, AGR62833.1, AGL68913.1, AGL68821.1, ADI34388.1/D6XNP4, ADI35260.1/D6XR66, AFJ82089.1, FJ81284.1	
aDDmanHepp	1-2	bLDmanHepp	GT9: AAD07549.1/O25224, AAX12679.1/Q5D6B0, ADZ50261.1, ADZ49368.1, ADZ51869.1, ADZ50969.1, AAD07342.1/O25056, AAD08237.1/O25802, AJF09417.1, AJF09505.1, AJF10958.1, AJF11046.1, ADU41556.1, ADU40654.1, ADU41461.1, ACX97491.1/D0IRE8, ACX98281.1/D0ITN8, ACX98900.1/D0JYI0, ACX99679.1/D0K0Q9, ACX99065.1/D0JYZ5, AEE70903.1, AEE70807.1, AEE69937.1, ADN79423.1/E1S7E3, ADN80404.1/E1S614, ADN80317.1/E1S9Y7, AFX91513.1, AFX90693.1, AFX89301.1, CAX28829.1/C7BYL9, CAX29815.1/C7C096, CBI65857.1/D7FCF0, CBI66840.1/D7FF83, AHA87976.1, AHA87527.1, AHA87687.1, AHZ27755.1, AHZ27907.1, AHA89259.1, AHA89548.1, AHA89098.1, AHZ27290.1, ADO04360.1/E1Q852, ADO03520.1/E1Q9P4, AFF20600.1, AFF20758.1, BAJ55726.1, BAJ54883.1, BAJ56298.1, BAJ57114.1, BAJ57870.1, BAJ58706.1, BAJ60228.1, BAJ59406.1, ACI27025.1/B5ZA45, ACI27886.1/B5Z8I7, ACI27201.1/B5Z6K2, ADU82017.1, ADU81160.1, AJD66044.1, AJD66128.1, ABF84348.1/Q1CUM4, ABF85199.1/Q1CS73, ABF84523.1/Q1CU49, AFI06987.1, AFI07806.1, ADU79509.1, ADU80449.1, AHZ25937.1, AHZ25850.1, AAD05843.1/Q9ZMF6, AAD06697.1/Q9ZK26, AAB65778.1/O30355, ADU82729.1, ADU83580.1, BAO97204.1, BAO97917.1, BAO97297.1, ADU82904.1, AAX12681.1/Q5D6A8, BAM96196.1, BAM97057.1, BAM98515.1, BAM97711.1, AHN35146.1, AHN36622.1, AHN37987.1, AHN39429.1, AHN40921.1, AHN42327.1, AHN43769.1, AHN45294.1, ACJ08309.1/B6JN31, ACJ07436.1/B6JKK8, AFI02069.1, AFI02915.1, ADO07374.1/E1Q3C6, ADO06530.1/E1Q539, AAX12680.1/Q5D6A9, AEN14924.1, AEN15757.1, AEN17991.1, AEN18885.1, AFV43096.1, AFV44002.1, AFV44690.1, AFV45595.1, ADO05028.1/E1PUI1, ADO05860.1/E1PWZ5, AFI01376.1, AFI00560.1, AFH99016.1, AFH99846.1, AFH98291.1, AFH97437.1, ACD48635.1/B2UUV3, ACD47742.1/B2USB0, ADO01898.1/E1PXZ9, ADO02755.1/E1Q0R4, AGL70824.1, AGL70916.1, AEN16487.1, AEN17320.1, AGT73551.1, AGT74495.1, ADU85141.1, ADU84307.1, AGL66809.1, AGL66716.1, AGL69452.1, AGL69358.1, AGL65939.1, AGR62916.1, AGR62833.1, AGL68913.1, AGL68821.1, ADI34388.1/D6XNP4, ADI35260.1/D6XR66, AFJ82089.1, FJ81284.1	
aDDmanHepp	1-2	aLDmanHepp	GT9: AAD07549.1/O25224, AAX12679.1/Q5D6B0, ADZ50261.1, ADZ49368.1, ADZ51869.1, ADZ50969.1, AAD07342.1/O25056, AAD08237.1/O25802, AJF09417.1, AJF09505.1, AJF10958.1, AJF11046.1, ADU41556.1, ADU40654.1, ADU41461.1, ACX97491.1/D0IRE8, ACX98281.1/D0ITN8, ACX98900.1/D0JYI0, ACX99679.1/D0K0Q9, ACX99065.1/D0JYZ5, AEE70903.1, AEE70807.1, AEE69937.1, ADN79423.1/E1S7E3, ADN80404.1/E1S614, ADN80317.1/E1S9Y7, AFX91513.1, AFX90693.1, AFX89301.1, CAX28829.1/C7BYL9, CAX29815.1/C7C096, CBI65857.1/D7FCF0, CBI66840.1/D7FF83, AHA87976.1, AHA87527.1, AHA87687.1, AHZ27755.1, AHZ27907.1, AHA89259.1, AHA89548.1, AHA89098.1, AHZ27290.1, ADO04360.1/E1Q852, ADO03520.1/E1Q9P4, AFF20600.1, AFF20758.1, BAJ55726.1, BAJ54883.1, BAJ56298.1, BAJ57114.1, BAJ57870.1, BAJ58706.1, BAJ60228.1, BAJ59406.1, ACI27025.1/B5ZA45, ACI27886.1/B5Z8I7, ACI27201.1/B5Z6K2, ADU82017.1, ADU81160.1, AJD66044.1, AJD66128.1, ABF84348.1/Q1CUM4, ABF85199.1/Q1CS73, ABF84523.1/Q1CU49, AFI06987.1, AFI07806.1, ADU79509.1, ADU80449.1, AHZ25937.1, AHZ25850.1, AAD05843.1/Q9ZMF6, AAD06697.1/Q9ZK26, AAB65778.1/O30355, ADU82729.1, ADU83580.1, BAO97204.1, BAO97917.1, BAO97297.1, ADU82904.1, AAX12681.1/Q5D6A8, BAM96196.1, BAM97057.1, BAM98515.1, BAM97711.1, AHN35146.1, AHN36622.1, AHN37987.1, AHN39429.1, AHN40921.1, AHN42327.1, AHN43769.1, AHN45294.1, ACJ08309.1/B6JN31, ACJ07436.1/B6JKK8, AFI02069.1, AFI02915.1, ADO07374.1/E1Q3C6, ADO06530.1/E1Q539, AAX12680.1/Q5D6A9, AEN14924.1, AEN15757.1, AEN17991.1, AEN18885.1, AFV43096.1, AFV44002.1, AFV44690.1, AFV45595.1, ADO05028.1/E1PUI1, ADO05860.1/E1PWZ5, AFI01376.1, AFI00560.1, AFH99016.1, AFH99846.1, AFH98291.1, AFH97437.1, ACD48635.1/B2UUV3, ACD47742.1/B2USB0, ADO01898.1/E1PXZ9, ADO02755.1/E1Q0R4, AGL70824.1, AGL70916.1, AEN16487.1, AEN17320.1, AGT73551.1, AGT74495.1, ADU85141.1, ADU84307.1, AGL66809.1, AGL66716.1, AGL69452.1, AGL69358.1, AGL65939.1, AGR62916.1, AGR62833.1, AGL68913.1, AGL68821.1, ADI34388.1/D6XNP4, ADI35260.1/D6XR66, AFJ82089.1, FJ81284.1	
aDDmanHepp	1-2	LDmanHepp	GT9: AAD07549.1/O25224, AAX12679.1/Q5D6B0, ADZ50261.1, ADZ49368.1, ADZ51869.1, ADZ50969.1, AAD07342.1/O25056, AAD08237.1/O25802, AJF09417.1, AJF09505.1, AJF10958.1, AJF11046.1, ADU41556.1, ADU40654.1, ADU41461.1, ACX97491.1/D0IRE8, ACX98281.1/D0ITN8, ACX98900.1/D0JYI0, ACX99679.1/D0K0Q9, ACX99065.1/D0JYZ5, AEE70903.1, AEE70807.1, AEE69937.1, ADN79423.1/E1S7E3, ADN80404.1/E1S614, ADN80317.1/E1S9Y7, AFX91513.1, AFX90693.1, AFX89301.1, CAX28829.1/C7BYL9, CAX29815.1/C7C096, CBI65857.1/D7FCF0, CBI66840.1/D7FF83, AHA87976.1, AHA87527.1, AHA87687.1, AHZ27755.1, AHZ27907.1, AHA89259.1, AHA89548.1, AHA89098.1, AHZ27290.1, ADO04360.1/E1Q852, ADO03520.1/E1Q9P4, AFF20600.1, AFF20758.1, BAJ55726.1, BAJ54883.1, BAJ56298.1, BAJ57114.1, BAJ57870.1, BAJ58706.1, BAJ60228.1, BAJ59406.1, ACI27025.1/B5ZA45, ACI27886.1/B5Z8I7, ACI27201.1/B5Z6K2, ADU82017.1, ADU81160.1, AJD66044.1, AJD66128.1, ABF84348.1/Q1CUM4, ABF85199.1/Q1CS73, ABF84523.1/Q1CU49, AFI06987.1, AFI07806.1, ADU79509.1, ADU80449.1, AHZ25937.1, AHZ25850.1, AAD05843.1/Q9ZMF6, AAD06697.1/Q9ZK26, AAB65778.1/O30355, ADU82729.1, ADU83580.1, BAO97204.1, BAO97917.1, BAO97297.1, ADU82904.1, AAX12681.1/Q5D6A8, BAM96196.1, BAM97057.1, BAM98515.1, BAM97711.1, AHN35146.1, AHN36622.1, AHN37987.1, AHN39429.1, AHN40921.1, AHN42327.1, AHN43769.1, AHN45294.1, ACJ08309.1/B6JN31, ACJ07436.1/B6JKK8, AFI02069.1, AFI02915.1, ADO07374.1/E1Q3C6, ADO06530.1/E1Q539, AAX12680.1/Q5D6A9, AEN14924.1, AEN15757.1, AEN17991.1, AEN18885.1, AFV43096.1, AFV44002.1, AFV44690.1, AFV45595.1, ADO05028.1/E1PUI1, ADO05860.1/E1PWZ5, AFI01376.1, AFI00560.1, AFH99016.1, AFH99846.1, AFH98291.1, AFH97437.1, ACD48635.1/B2UUV3, ACD47742.1/B2USB0, ADO01898.1/E1PXZ9, ADO02755.1/E1Q0R4, AGL70824.1, AGL70916.1, AEN16487.1, AEN17320.1, AGT73551.1, AGT74495.1, ADU85141.1, ADU84307.1, AGL66809.1, AGL66716.1, AGL69452.1, AGL69358.1, AGL65939.1, AGR62916.1, AGR62833.1, AGL68913.1, AGL68821.1, ADI34388.1/D6XNP4, ADI35260.1/D6XR66, AFJ82089.1, FJ81284.1	
aDDmanHepp	1-3	aDGlcp	GT9: AAX12679.1/Q5D6B0, ADZ50261.1, ADZ49368.1, ADZ51869.1, ADZ50969.1, AAD07342.1/O25056, AAD08237.1/O25802, AJF09417.1, AJF09505.1, AJF10958.1, AJF11046.1, ADU41556.1, ADU40654.1, ADU41461.1, ACX97491.1/D0IRE8, ACX98281.1/D0ITN8, ACX98900.1/D0JYI0, ACX99679.1/D0K0Q9, ACX99065.1/D0JYZ5, AEE70903.1, AEE70807.1, AEE69937.1, ADN79423.1/E1S7E3, ADN80404.1/E1S614, ADN80317.1/E1S9Y7, AFX91513.1, AFX90693.1, AFX89301.1, CAX28829.1/C7BYL9, CAX29815.1/C7C096, CBI65857.1/D7FCF0, CBI66840.1/D7FF83, AHA87976.1, AHA87527.1, AHA87687.1, AHZ27755.1, AHZ27907.1, AHA89259.1, AHA89548.1, AHA89098.1, AHZ27290.1, ADO04360.1/E1Q852, ADO03520.1/E1Q9P4, AFF20600.1, AFF20758.1, BAJ55726.1, BAJ54883.1, BAJ56298.1, BAJ57114.1, BAJ57870.1, BAJ58706.1, BAJ60228.1, BAJ59406.1, ACI27025.1/B5ZA45, ACI27886.1/B5Z8I7, ACI27201.1/B5Z6K2, ADU82017.1, ADU81160.1, AJD66044.1, AJD66128.1, ABF84348.1/Q1CUM4, ABF85199.1/Q1CS73, ABF84523.1/Q1CU49, AFI06987.1, AFI07806.1, ADU79509.1, ADU80449.1, AHZ25937.1, AHZ25850.1, AAD05843.1/Q9ZMF6, AAD06697.1/Q9ZK26, AAB65778.1/O30355, ADU82729.1, ADU83580.1, BAO97204.1, BAO97917.1, BAO97297.1, ADU82904.1, AAX12681.1/Q5D6A8, BAM96196.1, BAM97057.1, BAM98515.1, BAM97711.1, AHN35146.1, AHN36622.1, AHN37987.1, AHN39429.1, AHN40921.1, AHN42327.1, AHN43769.1, AHN45294.1, ACJ08309.1/B6JN31, ACJ07436.1/B6JKK8, AFI02069.1, AFI02915.1, ADO07374.1/E1Q3C6, ADO06530.1/E1Q539, AAX12680.1/Q5D6A9, AEN14924.1, AEN15757.1, AEN17991.1, AEN18885.1, AFV43096.1, AFV44002.1, AFV44690.1, AFV45595.1, ADO05028.1/E1PUI1, ADO05860.1/E1PWZ5, AFI01376.1, AFI00560.1, AFH99016.1, AFH99846.1, AFH98291.1, AFH97437.1, ACD48635.1/B2UUV3, ACD47742.1/B2USB0, ADO01898.1/E1PXZ9, ADO02755.1/E1Q0R4, AGL70824.1, AGL70916.1, AEN16487.1, AEN17320.1, AGT73551.1, AGT74495.1, ADU85141.1, ADU84307.1, AGL66809.1, AGL66716.1, AGL69452.1, AGL69358.1, AGL65939.1, AGR62916.1, AGR62833.1, AGL68913.1, AGL68821.1, ADI34388.1/D6XNP4, ADI35260.1/D6XR66, AFJ82089.1, FJ81284.1	
aDDmanHepp	1-3	aDDmanHepp	GT9: AAX12679.1/Q5D6B0, ADZ50261.1, ADZ49368.1, ADZ51869.1, ADZ50969.1, AAD07342.1/O25056, AAD08237.1/O25802, AJF09417.1, AJF09505.1, AJF10958.1, AJF11046.1, ADU41556.1, ADU40654.1, ADU41461.1, ACX97491.1/D0IRE8, ACX98281.1/D0ITN8, ACX98900.1/D0JYI0, ACX99679.1/D0K0Q9, ACX99065.1/D0JYZ5, AEE70903.1, AEE70807.1, AEE69937.1, ADN79423.1/E1S7E3, ADN80404.1/E1S614, ADN80317.1/E1S9Y7, AFX91513.1, AFX90693.1, AFX89301.1, CAX28829.1/C7BYL9, CAX29815.1/C7C096, CBI65857.1/D7FCF0, CBI66840.1/D7FF83, AHA87976.1, AHA87527.1, AHA87687.1, AHZ27755.1, AHZ27907.1, AHA89259.1, AHA89548.1, AHA89098.1, AHZ27290.1, ADO04360.1/E1Q852, ADO03520.1/E1Q9P4, AFF20600.1, AFF20758.1, BAJ55726.1, BAJ54883.1, BAJ56298.1, BAJ57114.1, BAJ57870.1, BAJ58706.1, BAJ60228.1, BAJ59406.1, ACI27025.1/B5ZA45, ACI27886.1/B5Z8I7, ACI27201.1/B5Z6K2, ADU82017.1, ADU81160.1, AJD66044.1, AJD66128.1, ABF84348.1/Q1CUM4, ABF85199.1/Q1CS73, ABF84523.1/Q1CU49, AFI06987.1, AFI07806.1, ADU79509.1, ADU80449.1, AHZ25937.1, AHZ25850.1, AAD05843.1/Q9ZMF6, AAD06697.1/Q9ZK26, AAB65778.1/O30355, ADU82729.1, ADU83580.1, BAO97204.1, BAO97917.1, BAO97297.1, ADU82904.1, AAX12681.1/Q5D6A8, BAM96196.1, BAM97057.1, BAM98515.1, BAM97711.1, AHN35146.1, AHN36622.1, AHN37987.1, AHN39429.1, AHN40921.1, AHN42327.1, AHN43769.1, AHN45294.1, ACJ08309.1/B6JN31, ACJ07436.1/B6JKK8, AFI02069.1, AFI02915.1, ADO07374.1/E1Q3C6, ADO06530.1/E1Q539, AAX12680.1/Q5D6A9, AEN14924.1, AEN15757.1, AEN17991.1, AEN18885.1, AFV43096.1, AFV44002.1, AFV44690.1, AFV45595.1, ADO05028.1/E1PUI1, ADO05860.1/E1PWZ5, AFI01376.1, AFI00560.1, AFH99016.1, AFH99846.1, AFH98291.1, AFH97437.1, ACD48635.1/B2UUV3, ACD47742.1/B2USB0, ADO01898.1/E1PXZ9, ADO02755.1/E1Q0R4, AGL70824.1, AGL70916.1, AEN16487.1, AEN17320.1, AGT73551.1, AGT74495.1, ADU85141.1, ADU84307.1, AGL66809.1, AGL66716.1, AGL69452.1, AGL69358.1, AGL65939.1, AGR62916.1, AGR62833.1, AGL68913.1, AGL68821.1, ADI34388.1/D6XNP4, ADI35260.1/D6XR66, AFJ82089.1, FJ81284.1	
aDDmanHepp	1-3	aLFucp	GT9: AAX12679.1/Q5D6B0, ADZ50261.1, ADZ49368.1, ADZ51869.1, ADZ50969.1, AAD07342.1/O25056, AAD08237.1/O25802, AJF09417.1, AJF09505.1, AJF10958.1, AJF11046.1, ADU41556.1, ADU40654.1, ADU41461.1, ACX97491.1/D0IRE8, ACX98281.1/D0ITN8, ACX98900.1/D0JYI0, ACX99679.1/D0K0Q9, ACX99065.1/D0JYZ5, AEE70903.1, AEE70807.1, AEE69937.1, ADN79423.1/E1S7E3, ADN80404.1/E1S614, ADN80317.1/E1S9Y7, AFX91513.1, AFX90693.1, AFX89301.1, CAX28829.1/C7BYL9, CAX29815.1/C7C096, CBI65857.1/D7FCF0, CBI66840.1/D7FF83, AHA87976.1, AHA87527.1, AHA87687.1, AHZ27755.1, AHZ27907.1, AHA89259.1, AHA89548.1, AHA89098.1, AHZ27290.1, ADO04360.1/E1Q852, ADO03520.1/E1Q9P4, AFF20600.1, AFF20758.1, BAJ55726.1, BAJ54883.1, BAJ56298.1, BAJ57114.1, BAJ57870.1, BAJ58706.1, BAJ60228.1, BAJ59406.1, ACI27025.1/B5ZA45, ACI27886.1/B5Z8I7, ACI27201.1/B5Z6K2, ADU82017.1, ADU81160.1, AJD66044.1, AJD66128.1, ABF84348.1/Q1CUM4, ABF85199.1/Q1CS73, ABF84523.1/Q1CU49, AFI06987.1, AFI07806.1, ADU79509.1, ADU80449.1, AHZ25937.1, AHZ25850.1, AAD05843.1/Q9ZMF6, AAD06697.1/Q9ZK26, AAB65778.1/O30355, ADU82729.1, ADU83580.1, BAO97204.1, BAO97917.1, BAO97297.1, ADU82904.1, AAX12681.1/Q5D6A8, BAM96196.1, BAM97057.1, BAM98515.1, BAM97711.1, AHN35146.1, AHN36622.1, AHN37987.1, AHN39429.1, AHN40921.1, AHN42327.1, AHN43769.1, AHN45294.1, ACJ08309.1/B6JN31, ACJ07436.1/B6JKK8, AFI02069.1, AFI02915.1, ADO07374.1/E1Q3C6, ADO06530.1/E1Q539, AAX12680.1/Q5D6A9, AEN14924.1, AEN15757.1, AEN17991.1, AEN18885.1, AFV43096.1, AFV44002.1, AFV44690.1, AFV45595.1, ADO05028.1/E1PUI1, ADO05860.1/E1PWZ5, AFI01376.1, AFI00560.1, AFH99016.1, AFH99846.1, AFH98291.1, AFH97437.1, ACD48635.1/B2UUV3, ACD47742.1/B2USB0, ADO01898.1/E1PXZ9, ADO02755.1/E1Q0R4, AGL70824.1, AGL70916.1, AEN16487.1, AEN17320.1, AGT73551.1, AGT74495.1, ADU85141.1, ADU84307.1, AGL66809.1, AGL66716.1, AGL69452.1, AGL69358.1, AGL65939.1, AGR62916.1, AGR62833.1, AGL68913.1, AGL68821.1, ADI34388.1/D6XNP4, ADI35260.1/D6XR66, AFJ82089.1, FJ81284.1	
aDDmanHepp	1-6	aDDmanHepp	GT9: AAX12679.1/Q5D6B0, ADZ50261.1, ADZ49368.1, ADZ51869.1, ADZ50969.1, AAD07342.1/O25056, AAD08237.1/O25802, AJF09417.1, AJF09505.1, AJF10958.1, AJF11046.1, ADU41556.1, ADU40654.1, ADU41461.1, ACX97491.1/D0IRE8, ACX98281.1/D0ITN8, ACX98900.1/D0JYI0, ACX99679.1/D0K0Q9, ACX99065.1/D0JYZ5, AEE70903.1, AEE70807.1, AEE69937.1, ADN79423.1/E1S7E3, ADN80404.1/E1S614, ADN80317.1/E1S9Y7, AFX91513.1, AFX90693.1, AFX89301.1, CAX28829.1/C7BYL9, CAX29815.1/C7C096, CBI65857.1/D7FCF0, CBI66840.1/D7FF83, AHA87976.1, AHA87527.1, AHA87687.1, AHZ27755.1, AHZ27907.1, AHA89259.1, AHA89548.1, AHA89098.1, AHZ27290.1, ADO04360.1/E1Q852, ADO03520.1/E1Q9P4, AFF20600.1, AFF20758.1, BAJ55726.1, BAJ54883.1, BAJ56298.1, BAJ57114.1, BAJ57870.1, BAJ58706.1, BAJ60228.1, BAJ59406.1, ACI27025.1/B5ZA45, ACI27886.1/B5Z8I7, ACI27201.1/B5Z6K2, ADU82017.1, ADU81160.1, AJD66044.1, AJD66128.1, ABF84348.1/Q1CUM4, ABF85199.1/Q1CS73, ABF84523.1/Q1CU49, AFI06987.1, AFI07806.1, ADU79509.1, ADU80449.1, AHZ25937.1, AHZ25850.1, AAD05843.1/Q9ZMF6, AAD06697.1/Q9ZK26, AAB65778.1/O30355, ADU82729.1, ADU83580.1, BAO97204.1, BAO97917.1, BAO97297.1, ADU82904.1, AAX12681.1/Q5D6A8, BAM96196.1, BAM97057.1, BAM98515.1, BAM97711.1, AHN35146.1, AHN36622.1, AHN37987.1, AHN39429.1, AHN40921.1, AHN42327.1, AHN43769.1, AHN45294.1, ACJ08309.1/B6JN31, ACJ07436.1/B6JKK8, AFI02069.1, AFI02915.1, ADO07374.1/E1Q3C6, ADO06530.1/E1Q539, AAX12680.1/Q5D6A9, AEN14924.1, AEN15757.1, AEN17991.1, AEN18885.1, AFV43096.1, AFV44002.1, AFV44690.1, AFV45595.1, ADO05028.1/E1PUI1, ADO05860.1/E1PWZ5, AFI01376.1, AFI00560.1, AFH99016.1, AFH99846.1, AFH98291.1, AFH97437.1, ACD48635.1/B2UUV3, ACD47742.1/B2USB0, ADO01898.1/E1PXZ9, ADO02755.1/E1Q0R4, AGL70824.1, AGL70916.1, AEN16487.1, AEN17320.1, AGT73551.1, AGT74495.1, ADU85141.1, ADU84307.1, AGL66809.1, AGL66716.1, AGL69452.1, AGL69358.1, AGL65939.1, AGR62916.1, AGR62833.1, AGL68913.1, AGL68821.1, ADI34388.1/D6XNP4, ADI35260.1/D6XR66, AFJ82089.1, FJ81284.1	
aDDmanHepp	1-7	aDDmanHepp	GT9: AAX12679.1/Q5D6B0, ADZ50261.1, ADZ49368.1, ADZ51869.1, ADZ50969.1, AAD07342.1/O25056, AAD08237.1/O25802, AJF09417.1, AJF09505.1, AJF10958.1, AJF11046.1, ADU41556.1, ADU40654.1, ADU41461.1, ACX97491.1/D0IRE8, ACX98281.1/D0ITN8, ACX98900.1/D0JYI0, ACX99679.1/D0K0Q9, ACX99065.1/D0JYZ5, AEE70903.1, AEE70807.1, AEE69937.1, ADN79423.1/E1S7E3, ADN80404.1/E1S614, ADN80317.1/E1S9Y7, AFX91513.1, AFX90693.1, AFX89301.1, CAX28829.1/C7BYL9, CAX29815.1/C7C096, CBI65857.1/D7FCF0, CBI66840.1/D7FF83, AHA87976.1, AHA87527.1, AHA87687.1, AHZ27755.1, AHZ27907.1, AHA89259.1, AHA89548.1, AHA89098.1, AHZ27290.1, ADO04360.1/E1Q852, ADO03520.1/E1Q9P4, AFF20600.1, AFF20758.1, BAJ55726.1, BAJ54883.1, BAJ56298.1, BAJ57114.1, BAJ57870.1, BAJ58706.1, BAJ60228.1, BAJ59406.1, ACI27025.1/B5ZA45, ACI27886.1/B5Z8I7, ACI27201.1/B5Z6K2, ADU82017.1, ADU81160.1, AJD66044.1, AJD66128.1, ABF84348.1/Q1CUM4, ABF85199.1/Q1CS73, ABF84523.1/Q1CU49, AFI06987.1, AFI07806.1, ADU79509.1, ADU80449.1, AHZ25937.1, AHZ25850.1, AAD05843.1/Q9ZMF6, AAD06697.1/Q9ZK26, AAB65778.1/O30355, ADU82729.1, ADU83580.1, BAO97204.1, BAO97917.1, BAO97297.1, ADU82904.1, AAX12681.1/Q5D6A8, BAM96196.1, BAM97057.1, BAM98515.1, BAM97711.1, AHN35146.1, AHN36622.1, AHN37987.1, AHN39429.1, AHN40921.1, AHN42327.1, AHN43769.1, AHN45294.1, ACJ08309.1/B6JN31, ACJ07436.1/B6JKK8, AFI02069.1, AFI02915.1, ADO07374.1/E1Q3C6, ADO06530.1/E1Q539, AAX12680.1/Q5D6A9, AEN14924.1, AEN15757.1, AEN17991.1, AEN18885.1, AFV43096.1, AFV44002.1, AFV44690.1, AFV45595.1, ADO05028.1/E1PUI1, ADO05860.1/E1PWZ5, AFI01376.1, AFI00560.1, AFH99016.1, AFH99846.1, AFH98291.1, AFH97437.1, ACD48635.1/B2UUV3, ACD47742.1/B2USB0, ADO01898.1/E1PXZ9, ADO02755.1/E1Q0R4, AGL70824.1, AGL70916.1, AEN16487.1, AEN17320.1, AGT73551.1, AGT74495.1, ADU85141.1, ADU84307.1, AGL66809.1, AGL66716.1, AGL69452.1, AGL69358.1, AGL65939.1, AGR62916.1, AGR62833.1, AGL68913.1, AGL68821.1, ADI34388.1/D6XNP4, ADI35260.1/D6XR66, AFJ82089.1, FJ81284.1	
aLDmanHepp	1-3	aLDmanHepp	GT9: ADZ50261.1, ADZ49368.1, ADZ51869.1, ADZ50969.1, AAD07342.1/O25056, AAD08237.1/O25802, AJF09417.1, AJF09505.1, AJF10958.1, AJF11046.1, ADU41556.1, ADU40654.1, ADU41461.1, ACX97491.1/D0IRE8, ACX98281.1/D0ITN8, ACX98900.1/D0JYI0, ACX99679.1/D0K0Q9, AEE70903.1, AEE70807.1, AEE69937.1, ADN79423.1/E1S7E3, ADN80404.1/E1S614, ADN80317.1/E1S9Y7, AFX91513.1, AFX90693.1, AFX89301.1, CAX28829.1/C7BYL9, CAX29815.1/C7C096, CBI65857.1/D7FCF0, CBI66840.1/D7FF83, AHA87976.1, AHA87527.1, AHA87687.1, AHZ27755.1, AHZ27907.1, AHA89259.1, AHA89548.1, AHA89098.1, AHZ27290.1, ADO04360.1/E1Q852, ADO03520.1/E1Q9P4, AFF20600.1, AFF20758.1, BAJ55726.1, BAJ54883.1, BAJ56298.1, BAJ57114.1, BAJ57870.1, BAJ58706.1, BAJ60228.1, BAJ59406.1, ACI27025.1/B5ZA45, ACI27886.1/B5Z8I7, ADU82017.1, ADU81160.1, AJD66044.1, AJD66128.1, ABF84348.1/Q1CUM4, ABF85199.1/Q1CS73, AFI06987.1, AFI07806.1, ADU79509.1, ADU80449.1, AHZ25937.1, AHZ25850.1, AAD05843.1/Q9ZMF6, AAD06697.1/Q9ZK26, AAB65778.1/O30355, ADU82729.1, ADU83580.1, BAO97204.1, BAO97917.1, BAO97297.1, BAM96196.1, BAM97057.1, BAM98515.1, BAM97711.1, AHN35146.1, AHN36622.1, AHN37987.1, AHN39429.1, AHN40921.1, AHN42327.1, AHN43769.1, AHN45294.1, ACJ08309.1/B6JN31, ACJ07436.1/B6JKK8, AFI02069.1, AFI02915.1, ADO07374.1/E1Q3C6, ADO06530.1/E1Q539, AEN14924.1, AEN15757.1, AEN17991.1, AEN18885.1, AFV43096.1, AFV44002.1, AFV44690.1, AFV45595.1, ADO05028.1/E1PUI1, ADO05860.1/E1PWZ5, AFI01376.1, AFI00560.1, AFH99016.1, AFH99846.1, AFH98291.1, AFH97437.1, ACD48635.1/B2UUV3, ACD47742.1/B2USB0, ADO01898.1/E1PXZ9, ADO02755.1/E1Q0R4, AGL70824.1, AGL70916.1, AEN16487.1, AEN17320.1, AGT73551.1, AGT74495.1, ADU85141.1, ADU84307.1, AGL66809.1, AGL66716.1, AGL69452.1, AGL69358.1, AGR62916.1, AGR62833.1, AGL68913.1, AGL68821.1, ADI34388.1/D6XNP4, ADI35260.1/D6XR66, AFJ82089.1, FJ81284.1	
aLDmanHepp	1-5	aKdop	GT9: ADZ50261.1, ADZ49368.1, ADZ51869.1, ADZ50969.1, AAD07342.1/O25056, AAD08237.1/O25802, AJF09417.1, AJF09505.1, AJF10958.1, AJF11046.1, ADU41556.1, ADU40654.1, ADU41461.1, ACX97491.1/D0IRE8, ACX98281.1/D0ITN8, ACX98900.1/D0JYI0, ACX99679.1/D0K0Q9, AEE70903.1, AEE70807.1, AEE69937.1, ADN79423.1/E1S7E3, ADN80404.1/E1S614, ADN80317.1/E1S9Y7, AFX91513.1, AFX90693.1, AFX89301.1, CAX28829.1/C7BYL9, CAX29815.1/C7C096, CBI65857.1/D7FCF0, CBI66840.1/D7FF83, AHA87976.1, AHA87527.1, AHA87687.1, AHZ27755.1, AHZ27907.1, AHA89259.1, AHA89548.1, AHA89098.1, AHZ27290.1, ADO04360.1/E1Q852, ADO03520.1/E1Q9P4, AFF20600.1, AFF20758.1, BAJ55726.1, BAJ54883.1, BAJ56298.1, BAJ57114.1, BAJ57870.1, BAJ58706.1, BAJ60228.1, BAJ59406.1, ACI27025.1/B5ZA45, ACI27886.1/B5Z8I7, ADU82017.1, ADU81160.1, AJD66044.1, AJD66128.1, ABF84348.1/Q1CUM4, ABF85199.1/Q1CS73, AFI06987.1, AFI07806.1, ADU79509.1, ADU80449.1, AHZ25937.1, AHZ25850.1, AAD05843.1/Q9ZMF6, AAD06697.1/Q9ZK26, AAB65778.1/O30355, ADU82729.1, ADU83580.1, BAO97204.1, BAO97917.1, BAO97297.1, BAM96196.1, BAM97057.1, BAM98515.1, BAM97711.1, AHN35146.1, AHN36622.1, AHN37987.1, AHN39429.1, AHN40921.1, AHN42327.1, AHN43769.1, AHN45294.1, ACJ08309.1/B6JN31, ACJ07436.1/B6JKK8, AFI02069.1, AFI02915.1, ADO07374.1/E1Q3C6, ADO06530.1/E1Q539, AEN14924.1, AEN15757.1, AEN17991.1, AEN18885.1, AFV43096.1, AFV44002.1, AFV44690.1, AFV45595.1, ADO05028.1/E1PUI1, ADO05860.1/E1PWZ5, AFI01376.1, AFI00560.1, AFH99016.1, AFH99846.1, AFH98291.1, AFH97437.1, ACD48635.1/B2UUV3, ACD47742.1/B2USB0, ADO01898.1/E1PXZ9, ADO02755.1/E1Q0R4, AGL70824.1, AGL70916.1, AEN16487.1, AEN17320.1, AGT73551.1, AGT74495.1, ADU85141.1, ADU84307.1, AGL66809.1, AGL66716.1, AGL69452.1, AGL69358.1, AGR62916.1, AGR62833.1, AGL68913.1, AGL68821.1, ADI34388.1/D6XNP4, ADI35260.1/D6XR66, AFJ82089.1, FJ81284.1	
aLDmanHepp	1-5	aKdo	GT9: ADZ50261.1, ADZ49368.1, ADZ51869.1, ADZ50969.1, AAD07342.1/O25056, AAD08237.1/O25802, AJF09417.1, AJF09505.1, AJF10958.1, AJF11046.1, ADU41556.1, ADU40654.1, ADU41461.1, ACX97491.1/D0IRE8, ACX98281.1/D0ITN8, ACX98900.1/D0JYI0, ACX99679.1/D0K0Q9, AEE70903.1, AEE70807.1, AEE69937.1, ADN79423.1/E1S7E3, ADN80404.1/E1S614, ADN80317.1/E1S9Y7, AFX91513.1, AFX90693.1, AFX89301.1, CAX28829.1/C7BYL9, CAX29815.1/C7C096, CBI65857.1/D7FCF0, CBI66840.1/D7FF83, AHA87976.1, AHA87527.1, AHA87687.1, AHZ27755.1, AHZ27907.1, AHA89259.1, AHA89548.1, AHA89098.1, AHZ27290.1, ADO04360.1/E1Q852, ADO03520.1/E1Q9P4, AFF20600.1, AFF20758.1, BAJ55726.1, BAJ54883.1, BAJ56298.1, BAJ57114.1, BAJ57870.1, BAJ58706.1, BAJ60228.1, BAJ59406.1, ACI27025.1/B5ZA45, ACI27886.1/B5Z8I7, ADU82017.1, ADU81160.1, AJD66044.1, AJD66128.1, ABF84348.1/Q1CUM4, ABF85199.1/Q1CS73, AFI06987.1, AFI07806.1, ADU79509.1, ADU80449.1, AHZ25937.1, AHZ25850.1, AAD05843.1/Q9ZMF6, AAD06697.1/Q9ZK26, AAB65778.1/O30355, ADU82729.1, ADU83580.1, BAO97204.1, BAO97917.1, BAO97297.1, BAM96196.1, BAM97057.1, BAM98515.1, BAM97711.1, AHN35146.1, AHN36622.1, AHN37987.1, AHN39429.1, AHN40921.1, AHN42327.1, AHN43769.1, AHN45294.1, ACJ08309.1/B6JN31, ACJ07436.1/B6JKK8, AFI02069.1, AFI02915.1, ADO07374.1/E1Q3C6, ADO06530.1/E1Q539, AEN14924.1, AEN15757.1, AEN17991.1, AEN18885.1, AFV43096.1, AFV44002.1, AFV44690.1, AFV45595.1, ADO05028.1/E1PUI1, ADO05860.1/E1PWZ5, AFI01376.1, AFI00560.1, AFH99016.1, AFH99846.1, AFH98291.1, AFH97437.1, ACD48635.1/B2UUV3, ACD47742.1/B2USB0, ADO01898.1/E1PXZ9, ADO02755.1/E1Q0R4, AGL70824.1, AGL70916.1, AEN16487.1, AEN17320.1, AGT73551.1, AGT74495.1, ADU85141.1, ADU84307.1, AGL66809.1, AGL66716.1, AGL69452.1, AGL69358.1, AGR62916.1, AGR62833.1, AGL68913.1, AGL68821.1, ADI34388.1/D6XNP4, ADI35260.1/D6XR66, AFJ82089.1, FJ81284.1	
aLDmanHepp	1-5	Kdo	GT9: ADZ50261.1, ADZ49368.1, ADZ51869.1, ADZ50969.1, AAD07342.1/O25056, AAD08237.1/O25802, AJF09417.1, AJF09505.1, AJF10958.1, AJF11046.1, ADU41556.1, ADU40654.1, ADU41461.1, ACX97491.1/D0IRE8, ACX98281.1/D0ITN8, ACX98900.1/D0JYI0, ACX99679.1/D0K0Q9, AEE70903.1, AEE70807.1, AEE69937.1, ADN79423.1/E1S7E3, ADN80404.1/E1S614, ADN80317.1/E1S9Y7, AFX91513.1, AFX90693.1, AFX89301.1, CAX28829.1/C7BYL9, CAX29815.1/C7C096, CBI65857.1/D7FCF0, CBI66840.1/D7FF83, AHA87976.1, AHA87527.1, AHA87687.1, AHZ27755.1, AHZ27907.1, AHA89259.1, AHA89548.1, AHA89098.1, AHZ27290.1, ADO04360.1/E1Q852, ADO03520.1/E1Q9P4, AFF20600.1, AFF20758.1, BAJ55726.1, BAJ54883.1, BAJ56298.1, BAJ57114.1, BAJ57870.1, BAJ58706.1, BAJ60228.1, BAJ59406.1, ACI27025.1/B5ZA45, ACI27886.1/B5Z8I7, ADU82017.1, ADU81160.1, AJD66044.1, AJD66128.1, ABF84348.1/Q1CUM4, ABF85199.1/Q1CS73, AFI06987.1, AFI07806.1, ADU79509.1, ADU80449.1, AHZ25937.1, AHZ25850.1, AAD05843.1/Q9ZMF6, AAD06697.1/Q9ZK26, AAB65778.1/O30355, ADU82729.1, ADU83580.1, BAO97204.1, BAO97917.1, BAO97297.1, BAM96196.1, BAM97057.1, BAM98515.1, BAM97711.1, AHN35146.1, AHN36622.1, AHN37987.1, AHN39429.1, AHN40921.1, AHN42327.1, AHN43769.1, AHN45294.1, ACJ08309.1/B6JN31, ACJ07436.1/B6JKK8, AFI02069.1, AFI02915.1, ADO07374.1/E1Q3C6, ADO06530.1/E1Q539, AEN14924.1, AEN15757.1, AEN17991.1, AEN18885.1, AFV43096.1, AFV44002.1, AFV44690.1, AFV45595.1, ADO05028.1/E1PUI1, ADO05860.1/E1PWZ5, AFI01376.1, AFI00560.1, AFH99016.1, AFH99846.1, AFH98291.1, AFH97437.1, ACD48635.1/B2UUV3, ACD47742.1/B2USB0, ADO01898.1/E1PXZ9, ADO02755.1/E1Q0R4, AGL70824.1, AGL70916.1, AEN16487.1, AEN17320.1, AGT73551.1, AGT74495.1, ADU85141.1, ADU84307.1, AGL66809.1, AGL66716.1, AGL69452.1, AGL69358.1, AGR62916.1, AGR62833.1, AGL68913.1, AGL68821.1, ADI34388.1/D6XNP4, ADI35260.1/D6XR66, AFJ82089.1, FJ81284.1	
bLDmanHepp	1-3	aLDmanHepp	GT9: ADZ50261.1, ADZ49368.1, ADZ51869.1, ADZ50969.1, AAD07342.1/O25056, AAD08237.1/O25802, AJF09417.1, AJF09505.1, AJF10958.1, AJF11046.1, ADU41556.1, ADU40654.1, ADU41461.1, ACX97491.1/D0IRE8, ACX98281.1/D0ITN8, ACX98900.1/D0JYI0, ACX99679.1/D0K0Q9, AEE70903.1, AEE70807.1, AEE69937.1, ADN79423.1/E1S7E3, ADN80404.1/E1S614, ADN80317.1/E1S9Y7, AFX91513.1, AFX90693.1, AFX89301.1, CAX28829.1/C7BYL9, CAX29815.1/C7C096, CBI65857.1/D7FCF0, CBI66840.1/D7FF83, AHA87976.1, AHA87527.1, AHA87687.1, AHZ27755.1, AHZ27907.1, AHA89259.1, AHA89548.1, AHA89098.1, AHZ27290.1, ADO04360.1/E1Q852, ADO03520.1/E1Q9P4, AFF20600.1, AFF20758.1, BAJ55726.1, BAJ54883.1, BAJ56298.1, BAJ57114.1, BAJ57870.1, BAJ58706.1, BAJ60228.1, BAJ59406.1, ACI27025.1/B5ZA45, ACI27886.1/B5Z8I7, ADU82017.1, ADU81160.1, AJD66044.1, AJD66128.1, ABF84348.1/Q1CUM4, ABF85199.1/Q1CS73, AFI06987.1, AFI07806.1, ADU79509.1, ADU80449.1, AHZ25937.1, AHZ25850.1, AAD05843.1/Q9ZMF6, AAD06697.1/Q9ZK26, AAB65778.1/O30355, ADU82729.1, ADU83580.1, BAO97204.1, BAO97917.1, BAO97297.1, BAM96196.1, BAM97057.1, BAM98515.1, BAM97711.1, AHN35146.1, AHN36622.1, AHN37987.1, AHN39429.1, AHN40921.1, AHN42327.1, AHN43769.1, AHN45294.1, ACJ08309.1/B6JN31, ACJ07436.1/B6JKK8, AFI02069.1, AFI02915.1, ADO07374.1/E1Q3C6, ADO06530.1/E1Q539, AEN14924.1, AEN15757.1, AEN17991.1, AEN18885.1, AFV43096.1, AFV44002.1, AFV44690.1, AFV45595.1, ADO05028.1/E1PUI1, ADO05860.1/E1PWZ5, AFI01376.1, AFI00560.1, AFH99016.1, AFH99846.1, AFH98291.1, AFH97437.1, ACD48635.1/B2UUV3, ACD47742.1/B2USB0, ADO01898.1/E1PXZ9, ADO02755.1/E1Q0R4, AGL70824.1, AGL70916.1, AEN16487.1, AEN17320.1, AGT73551.1, AGT74495.1, ADU85141.1, ADU84307.1, AGL66809.1, AGL66716.1, AGL69452.1, AGL69358.1, AGR62916.1, AGR62833.1, AGL68913.1, AGL68821.1, ADI34388.1/D6XNP4, ADI35260.1/D6XR66, AFJ82089.1, FJ81284.1	
LDmanHepp	1-5	Kdo	GT9: ADZ50261.1, ADZ49368.1, ADZ51869.1, ADZ50969.1, AAD07342.1/O25056, AAD08237.1/O25802, AJF09417.1, AJF09505.1, AJF10958.1, AJF11046.1, ADU41556.1, ADU40654.1, ADU41461.1, ACX97491.1/D0IRE8, ACX98281.1/D0ITN8, ACX98900.1/D0JYI0, ACX99679.1/D0K0Q9, AEE70903.1, AEE70807.1, AEE69937.1, ADN79423.1/E1S7E3, ADN80404.1/E1S614, ADN80317.1/E1S9Y7, AFX91513.1, AFX90693.1, AFX89301.1, CAX28829.1/C7BYL9, CAX29815.1/C7C096, CBI65857.1/D7FCF0, CBI66840.1/D7FF83, AHA87976.1, AHA87527.1, AHA87687.1, AHZ27755.1, AHZ27907.1, AHA89259.1, AHA89548.1, AHA89098.1, AHZ27290.1, ADO04360.1/E1Q852, ADO03520.1/E1Q9P4, AFF20600.1, AFF20758.1, BAJ55726.1, BAJ54883.1, BAJ56298.1, BAJ57114.1, BAJ57870.1, BAJ58706.1, BAJ60228.1, BAJ59406.1, ACI27025.1/B5ZA45, ACI27886.1/B5Z8I7, ADU82017.1, ADU81160.1, AJD66044.1, AJD66128.1, ABF84348.1/Q1CUM4, ABF85199.1/Q1CS73, AFI06987.1, AFI07806.1, ADU79509.1, ADU80449.1, AHZ25937.1, AHZ25850.1, AAD05843.1/Q9ZMF6, AAD06697.1/Q9ZK26, AAB65778.1/O30355, ADU82729.1, ADU83580.1, BAO97204.1, BAO97917.1, BAO97297.1, BAM96196.1, BAM97057.1, BAM98515.1, BAM97711.1, AHN35146.1, AHN36622.1, AHN37987.1, AHN39429.1, AHN40921.1, AHN42327.1, AHN43769.1, AHN45294.1, ACJ08309.1/B6JN31, ACJ07436.1/B6JKK8, AFI02069.1, AFI02915.1, ADO07374.1/E1Q3C6, ADO06530.1/E1Q539, AEN14924.1, AEN15757.1, AEN17991.1, AEN18885.1, AFV43096.1, AFV44002.1, AFV44690.1, AFV45595.1, ADO05028.1/E1PUI1, ADO05860.1/E1PWZ5, AFI01376.1, AFI00560.1, AFH99016.1, AFH99846.1, AFH98291.1, AFH97437.1, ACD48635.1/B2UUV3, ACD47742.1/B2USB0, ADO01898.1/E1PXZ9, ADO02755.1/E1Q0R4, AGL70824.1, AGL70916.1, AEN16487.1, AEN17320.1, AGT73551.1, AGT74495.1, ADU85141.1, ADU84307.1, AGL66809.1, AGL66716.1, AGL69452.1, AGL69358.1, AGR62916.1, AGR62833.1, AGL68913.1, AGL68821.1, ADI34388.1/D6XNP4, ADI35260.1/D6XR66, AFJ82089.1, FJ81284.1	
aDFucp	1-2	aDDmanHepp	GT11: AAD29863.1/Q9X435, AAC99764.1/Q9X3N7, ADZ49177.1, DZ49178.1, ADZ50777.1, AJF08410.1, AJF08411.1, AJF09954.1, AJF09955.1, ADN79234.1/E1S5X7, ABO61751.1/A4L7J2, CAX28646.1/C7BXZ3, CBI67029.1/D7FFS2, AHZ28011.1, AHZ28012.1, AHZ26262.1, AHZ29189.1, BAJ54704.1, BAJ54703.1, BAJ57270.1, BAJ57269.1, BAJ57688.1, BAJ57687.1, BAJ59215.1, AJD65149.1, AJD65150.1, ABF84161.1/Q1CV61, ABO61750.1/A4L7J1, AHZ24811.1, AAD05659.1/Q9ZMX9, ADU82546.1, BAO97720.1, BAO97721.1, AM96013.1, BAM96014.1, BAM97534.1, AHN34165.1, AHN38481.1, AHN41388.1, ACJ07256.1/B6JPJ5, AFV42906.1, AFV44501.1, AFI00328.1, ACD47556.1/B2URS4, ADU84121.1, AAD29865.1/Q9X436, AAD29868.1/Q9X440, AAD29869.1/Q9X441, AAD29867.1/Q9X439, AGL66039.2, AGL71980.1, AGR63312.1, AGS15597.1, AFJ81141.1	
aLFucp	1-2	bDGalp	GT11: AAD29863.1/Q9X435, AAC99764.1/Q9X3N7, ADZ49177.1, DZ49178.1, ADZ50777.1, AJF08410.1, AJF08411.1, AJF09954.1, AJF09955.1, ADN79234.1/E1S5X7, ABO61751.1/A4L7J2, CAX28646.1/C7BXZ3, CBI67029.1/D7FFS2, AHZ28011.1, AHZ28012.1, AHZ26262.1, AHZ29189.1, BAJ54704.1, BAJ54703.1, BAJ57270.1, BAJ57269.1, BAJ57688.1, BAJ57687.1, BAJ59215.1, AJD65149.1, AJD65150.1, ABF84161.1/Q1CV61, ABO61750.1/A4L7J1, AHZ24811.1, AAD05659.1/Q9ZMX9, ADU82546.1, BAO97720.1, BAO97721.1, AM96013.1, BAM96014.1, BAM97534.1, AHN34165.1, AHN38481.1, AHN41388.1, ACJ07256.1/B6JPJ5, AFV42906.1, AFV44501.1, AFI00328.1, ACD47556.1/B2URS4, ADU84121.1, AAD29865.1/Q9X436, AAD29868.1/Q9X440, AAD29869.1/Q9X441, AAD29867.1/Q9X439, AGL66039.2, AGL71980.1, AGR63312.1, AGS15597.1, AFJ81141.1	
aLFucp	1-2	bDGal	GT11: AAD29863.1/Q9X435, AAC99764.1/Q9X3N7, ADZ49177.1, DZ49178.1, ADZ50777.1, AJF08410.1, AJF08411.1, AJF09954.1, AJF09955.1, ADN79234.1/E1S5X7, ABO61751.1/A4L7J2, CAX28646.1/C7BXZ3, CBI67029.1/D7FFS2, AHZ28011.1, AHZ28012.1, AHZ26262.1, AHZ29189.1, BAJ54704.1, BAJ54703.1, BAJ57270.1, BAJ57269.1, BAJ57688.1, BAJ57687.1, BAJ59215.1, AJD65149.1, AJD65150.1, ABF84161.1/Q1CV61, ABO61750.1/A4L7J1, AHZ24811.1, AAD05659.1/Q9ZMX9, ADU82546.1, BAO97720.1, BAO97721.1, AM96013.1, BAM96014.1, BAM97534.1, AHN34165.1, AHN38481.1, AHN41388.1, ACJ07256.1/B6JPJ5, AFV42906.1, AFV44501.1, AFI00328.1, ACD47556.1/B2URS4, ADU84121.1, AAD29865.1/Q9X436, AAD29868.1/Q9X440, AAD29869.1/Q9X441, AAD29867.1/Q9X439, AGL66039.2, AGL71980.1, AGR63312.1, AGS15597.1, AFJ81141.1	
aLFucp	1-3	aDGlcpN	GT10: AAB93985.1/O32631, AAD06169.1/Q9ZLI3, AAD06573.1/Q9ZKD7, AAB81031.1/O30511, AAF35291.2/Q9L8S4, ABZ68520.1, ADZ49735.1, ADZ51338.1, AAD07447.1/O25142, AAD07710.1/O25366, ACX97875.1/D0ISI2, ACX98174.1/D0ITD1, ACX99573.1/D0K0F3, ADN79791.1/E1S8G1, ADN80190.1/E1S9L0, AFX91028.1, AFX91400.1, AFX89961.1, AFX90099.1, CAX29250.1/C7BZU7, CAX29559.1/C7BXF2, CBI65984.1/D7FCS7, AHA88301.1, AHA88720.1, AHA89874.1, AHA90294.1, ADO04239.1/E1Q7N0, ADO03869.1/E1QB24, AFF19835.1, AFF20202.1, BAJ55615.1, BAJ55257.1, BAJ56774.1, BAJ56409.1, BAJ57911.1, BAJ58250.1, BAJ59748.1, BAJ60112.1, ACI27373.1/B5Z724, ACI27771.1/B5Z872, ADU81508.1, ADU81895.1, ABF84703.1/Q1CTL9, ABF85080.1/Q1CSJ2, AFI07382.1, AFI07690.1, ADU83456.1, ADU83076.1, BAM96573.1, BAM96944.1, BAM98073.1, BAM98401.1, ACJ07817.1/B6JLN9, ACJ08194.1/B6JMR6, AFI02397.1, AFI02795.1, ADO07257.1/E1Q309, ADO06937.1/E1Q6J4, AEN15635.1, AEN15311.1, AEN18764.1, AEN18341.1, ADO05746.1/E1PWN1, ADO05445.1/E1PVT0, AFI00953.1, AFI01260.1, AFH99730.1, AFH98149.1, AFH97781.1, ACD48158.1/B2UTH6, ACD48513.1/B2UUI1, ADO02255.1/E1PZB4, ADO02630.1/E1Q0D9, AEN16830.1, ADU84619.1, ADU85018.1, AGL70321.1, AGL71396.1, AGL71039.1, ADI34738.1/D6XPP4, AFJ81975.1, AFJ81683.1	
aLFucp	1-3	bDGlcpN	GT10: AAB93985.1/O32631, AAD06169.1/Q9ZLI3, AAD06573.1/Q9ZKD7, AAB81031.1/O30511, AAF35291.2/Q9L8S4, ABZ68520.1, ADZ49735.1, ADZ51338.1, AAD07447.1/O25142, AAD07710.1/O25366, ACX97875.1/D0ISI2, ACX98174.1/D0ITD1, ACX99573.1/D0K0F3, ADN79791.1/E1S8G1, ADN80190.1/E1S9L0, AFX91028.1, AFX91400.1, AFX89961.1, AFX90099.1, CAX29250.1/C7BZU7, CAX29559.1/C7BXF2, CBI65984.1/D7FCS7, AHA88301.1, AHA88720.1, AHA89874.1, AHA90294.1, ADO04239.1/E1Q7N0, ADO03869.1/E1QB24, AFF19835.1, AFF20202.1, BAJ55615.1, BAJ55257.1, BAJ56774.1, BAJ56409.1, BAJ57911.1, BAJ58250.1, BAJ59748.1, BAJ60112.1, ACI27373.1/B5Z724, ACI27771.1/B5Z872, ADU81508.1, ADU81895.1, ABF84703.1/Q1CTL9, ABF85080.1/Q1CSJ2, AFI07382.1, AFI07690.1, ADU83456.1, ADU83076.1, BAM96573.1, BAM96944.1, BAM98073.1, BAM98401.1, ACJ07817.1/B6JLN9, ACJ08194.1/B6JMR6, AFI02397.1, AFI02795.1, ADO07257.1/E1Q309, ADO06937.1/E1Q6J4, AEN15635.1, AEN15311.1, AEN18764.1, AEN18341.1, ADO05746.1/E1PWN1, ADO05445.1/E1PVT0, AFI00953.1, AFI01260.1, AFH99730.1, AFH98149.1, AFH97781.1, ACD48158.1/B2UTH6, ACD48513.1/B2UUI1, ADO02255.1/E1PZB4, ADO02630.1/E1Q0D9, AEN16830.1, ADU84619.1, ADU85018.1, AGL70321.1, AGL71396.1, AGL71039.1, ADI34738.1/D6XPP4, AFJ81975.1, AFJ81683.1	
aLFucp	1-3	bDGlcN
(assumed bDGlcpN)	GT10: AAB93985.1/O32631, AAD06169.1/Q9ZLI3, AAD06573.1/Q9ZKD7, AAB81031.1/O30511, AAF35291.2/Q9L8S4, ABZ68520.1, ADZ49735.1, ADZ51338.1, AAD07447.1/O25142, AAD07710.1/O25366, ACX97875.1/D0ISI2, ACX98174.1/D0ITD1, ACX99573.1/D0K0F3, ADN79791.1/E1S8G1, ADN80190.1/E1S9L0, AFX91028.1, AFX91400.1, AFX89961.1, AFX90099.1, CAX29250.1/C7BZU7, CAX29559.1/C7BXF2, CBI65984.1/D7FCS7, AHA88301.1, AHA88720.1, AHA89874.1, AHA90294.1, ADO04239.1/E1Q7N0, ADO03869.1/E1QB24, AFF19835.1, AFF20202.1, BAJ55615.1, BAJ55257.1, BAJ56774.1, BAJ56409.1, BAJ57911.1, BAJ58250.1, BAJ59748.1, BAJ60112.1, ACI27373.1/B5Z724, ACI27771.1/B5Z872, ADU81508.1, ADU81895.1, ABF84703.1/Q1CTL9, ABF85080.1/Q1CSJ2, AFI07382.1, AFI07690.1, ADU83456.1, ADU83076.1, BAM96573.1, BAM96944.1, BAM98073.1, BAM98401.1, ACJ07817.1/B6JLN9, ACJ08194.1/B6JMR6, AFI02397.1, AFI02795.1, ADO07257.1/E1Q309, ADO06937.1/E1Q6J4, AEN15635.1, AEN15311.1, AEN18764.1, AEN18341.1, ADO05746.1/E1PWN1, ADO05445.1/E1PVT0, AFI00953.1, AFI01260.1, AFH99730.1, AFH98149.1, AFH97781.1, ACD48158.1/B2UTH6, ACD48513.1/B2UUI1, ADO02255.1/E1PZB4, ADO02630.1/E1Q0D9, AEN16830.1, ADU84619.1, ADU85018.1, AGL70321.1, AGL71396.1, AGL71039.1, ADI34738.1/D6XPP4, AFJ81975.1, AFJ81683.1	
aLFucp	1-3	DGlcpN	GT10: AAB93985.1/O32631, AAD06169.1/Q9ZLI3, AAD06573.1/Q9ZKD7, AAB81031.1/O30511, AAF35291.2/Q9L8S4, ABZ68520.1, ADZ49735.1, ADZ51338.1, AAD07447.1/O25142, AAD07710.1/O25366, ACX97875.1/D0ISI2, ACX98174.1/D0ITD1, ACX99573.1/D0K0F3, ADN79791.1/E1S8G1, ADN80190.1/E1S9L0, AFX91028.1, AFX91400.1, AFX89961.1, AFX90099.1, CAX29250.1/C7BZU7, CAX29559.1/C7BXF2, CBI65984.1/D7FCS7, AHA88301.1, AHA88720.1, AHA89874.1, AHA90294.1, ADO04239.1/E1Q7N0, ADO03869.1/E1QB24, AFF19835.1, AFF20202.1, BAJ55615.1, BAJ55257.1, BAJ56774.1, BAJ56409.1, BAJ57911.1, BAJ58250.1, BAJ59748.1, BAJ60112.1, ACI27373.1/B5Z724, ACI27771.1/B5Z872, ADU81508.1, ADU81895.1, ABF84703.1/Q1CTL9, ABF85080.1/Q1CSJ2, AFI07382.1, AFI07690.1, ADU83456.1, ADU83076.1, BAM96573.1, BAM96944.1, BAM98073.1, BAM98401.1, ACJ07817.1/B6JLN9, ACJ08194.1/B6JMR6, AFI02397.1, AFI02795.1, ADO07257.1/E1Q309, ADO06937.1/E1Q6J4, AEN15635.1, AEN15311.1, AEN18764.1, AEN18341.1, ADO05746.1/E1PWN1, ADO05445.1/E1PVT0, AFI00953.1, AFI01260.1, AFH99730.1, AFH98149.1, AFH97781.1, ACD48158.1/B2UTH6, ACD48513.1/B2UUI1, ADO02255.1/E1PZB4, ADO02630.1/E1Q0D9, AEN16830.1, ADU84619.1, ADU85018.1, AGL70321.1, AGL71396.1, AGL71039.1, ADI34738.1/D6XPP4, AFJ81975.1, AFJ81683.1	
aLFucp	1-3	DGlcN	GT10: AAB93985.1/O32631, AAD06169.1/Q9ZLI3, AAD06573.1/Q9ZKD7, AAB81031.1/O30511, AAF35291.2/Q9L8S4, ABZ68520.1, ADZ49735.1, ADZ51338.1, AAD07447.1/O25142, AAD07710.1/O25366, ACX97875.1/D0ISI2, ACX98174.1/D0ITD1, ACX99573.1/D0K0F3, ADN79791.1/E1S8G1, ADN80190.1/E1S9L0, AFX91028.1, AFX91400.1, AFX89961.1, AFX90099.1, CAX29250.1/C7BZU7, CAX29559.1/C7BXF2, CBI65984.1/D7FCS7, AHA88301.1, AHA88720.1, AHA89874.1, AHA90294.1, ADO04239.1/E1Q7N0, ADO03869.1/E1QB24, AFF19835.1, AFF20202.1, BAJ55615.1, BAJ55257.1, BAJ56774.1, BAJ56409.1, BAJ57911.1, BAJ58250.1, BAJ59748.1, BAJ60112.1, ACI27373.1/B5Z724, ACI27771.1/B5Z872, ADU81508.1, ADU81895.1, ABF84703.1/Q1CTL9, ABF85080.1/Q1CSJ2, AFI07382.1, AFI07690.1, ADU83456.1, ADU83076.1, BAM96573.1, BAM96944.1, BAM98073.1, BAM98401.1, ACJ07817.1/B6JLN9, ACJ08194.1/B6JMR6, AFI02397.1, AFI02795.1, ADO07257.1/E1Q309, ADO06937.1/E1Q6J4, AEN15635.1, AEN15311.1, AEN18764.1, AEN18341.1, ADO05746.1/E1PWN1, ADO05445.1/E1PVT0, AFI00953.1, AFI01260.1, AFH99730.1, AFH98149.1, AFH97781.1, ACD48158.1/B2UTH6, ACD48513.1/B2UUI1, ADO02255.1/E1PZB4, ADO02630.1/E1Q0D9, AEN16830.1, ADU84619.1, ADU85018.1, AGL70321.1, AGL71396.1, AGL71039.1, ADI34738.1/D6XPP4, AFJ81975.1, AFJ81683.1	
aLFucp	1-3	aLFucp	GT10: AAB93985.1/O32631, AAD06169.1/Q9ZLI3, AAD06573.1/Q9ZKD7, AAB81031.1/O30511, AAF35291.2/Q9L8S4, ABZ68520.1, ADZ49735.1, ADZ51338.1, AAD07447.1/O25142, AAD07710.1/O25366, ACX97875.1/D0ISI2, ACX98174.1/D0ITD1, ACX99573.1/D0K0F3, ADN79791.1/E1S8G1, ADN80190.1/E1S9L0, AFX91028.1, AFX91400.1, AFX89961.1, AFX90099.1, CAX29250.1/C7BZU7, CAX29559.1/C7BXF2, CBI65984.1/D7FCS7, AHA88301.1, AHA88720.1, AHA89874.1, AHA90294.1, ADO04239.1/E1Q7N0, ADO03869.1/E1QB24, AFF19835.1, AFF20202.1, BAJ55615.1, BAJ55257.1, BAJ56774.1, BAJ56409.1, BAJ57911.1, BAJ58250.1, BAJ59748.1, BAJ60112.1, ACI27373.1/B5Z724, ACI27771.1/B5Z872, ADU81508.1, ADU81895.1, ABF84703.1/Q1CTL9, ABF85080.1/Q1CSJ2, AFI07382.1, AFI07690.1, ADU83456.1, ADU83076.1, BAM96573.1, BAM96944.1, BAM98073.1, BAM98401.1, ACJ07817.1/B6JLN9, ACJ08194.1/B6JMR6, AFI02397.1, AFI02795.1, ADO07257.1/E1Q309, ADO06937.1/E1Q6J4, AEN15635.1, AEN15311.1, AEN18764.1, AEN18341.1, ADO05746.1/E1PWN1, ADO05445.1/E1PVT0, AFI00953.1, AFI01260.1, AFH99730.1, AFH98149.1, AFH97781.1, ACD48158.1/B2UTH6, ACD48513.1/B2UUI1, ADO02255.1/E1PZB4, ADO02630.1/E1Q0D9, AEN16830.1, ADU84619.1, ADU85018.1, AGL70321.1, AGL71396.1, AGL71039.1, ADI34738.1/D6XPP4, AFJ81975.1, AFJ81683.1	
aLFucp	1-4	bDGlcpN	GT10: AAF35291.2/Q9L8S4, AAR88243.1/Q6ST35, ABZ68520.1, AAD07447.1/O25142, AAD07710.1/O25366, AAR88243.1/Q6ST35, AGL71396.1, AGL71039.1	
aLFucp	1-4	bDGlcN
(assumed bDGlcpN)	GT10: AAF35291.2/Q9L8S4, AAR88243.1/Q6ST35, ABZ68520.1, AAD07447.1/O25142, AAD07710.1/O25366, AAR88243.1/Q6ST35, AGL71396.1, AGL71039.1	
aLFucp	1-4	DGlcN	GT10: AAF35291.2/Q9L8S4, AAR88243.1/Q6ST35, ABZ68520.1, AAD07447.1/O25142, AAD07710.1/O25366, AAR88243.1/Q6ST35, AGL71396.1, AGL71039.1	
aLFuc
(assumed aLFucp)	1-3	bDGlcpN	GT10: AAB93985.1/O32631, AAD06169.1/Q9ZLI3, AAD06573.1/Q9ZKD7, AAB81031.1/O30511, AAF35291.2/Q9L8S4, ABZ68520.1, ADZ49735.1, ADZ51338.1, AAD07447.1/O25142, AAD07710.1/O25366, ACX97875.1/D0ISI2, ACX98174.1/D0ITD1, ACX99573.1/D0K0F3, ADN79791.1/E1S8G1, ADN80190.1/E1S9L0, AFX91028.1, AFX91400.1, AFX89961.1, AFX90099.1, CAX29250.1/C7BZU7, CAX29559.1/C7BXF2, CBI65984.1/D7FCS7, AHA88301.1, AHA88720.1, AHA89874.1, AHA90294.1, ADO04239.1/E1Q7N0, ADO03869.1/E1QB24, AFF19835.1, AFF20202.1, BAJ55615.1, BAJ55257.1, BAJ56774.1, BAJ56409.1, BAJ57911.1, BAJ58250.1, BAJ59748.1, BAJ60112.1, ACI27373.1/B5Z724, ACI27771.1/B5Z872, ADU81508.1, ADU81895.1, ABF84703.1/Q1CTL9, ABF85080.1/Q1CSJ2, AFI07382.1, AFI07690.1, ADU83456.1, ADU83076.1, BAM96573.1, BAM96944.1, BAM98073.1, BAM98401.1, ACJ07817.1/B6JLN9, ACJ08194.1/B6JMR6, AFI02397.1, AFI02795.1, ADO07257.1/E1Q309, ADO06937.1/E1Q6J4, AEN15635.1, AEN15311.1, AEN18764.1, AEN18341.1, ADO05746.1/E1PWN1, ADO05445.1/E1PVT0, AFI00953.1, AFI01260.1, AFH99730.1, AFH98149.1, AFH97781.1, ACD48158.1/B2UTH6, ACD48513.1/B2UUI1, ADO02255.1/E1PZB4, ADO02630.1/E1Q0D9, AEN16830.1, ADU84619.1, ADU85018.1, AGL70321.1, AGL71396.1, AGL71039.1, ADI34738.1/D6XPP4, AFJ81975.1, AFJ81683.1	
aFuc	1-3	bGlcN	GT10: AAB93985.1/O32631, AAD06169.1/Q9ZLI3, AAD06573.1/Q9ZKD7, AAB81031.1/O30511, AAF35291.2/Q9L8S4, ABZ68520.1, ADZ49735.1, ADZ51338.1, AAD07447.1/O25142, AAD07710.1/O25366, ACX97875.1/D0ISI2, ACX98174.1/D0ITD1, ACX99573.1/D0K0F3, ADN79791.1/E1S8G1, ADN80190.1/E1S9L0, AFX91028.1, AFX91400.1, AFX89961.1, AFX90099.1, CAX29250.1/C7BZU7, CAX29559.1/C7BXF2, CBI65984.1/D7FCS7, AHA88301.1, AHA88720.1, AHA89874.1, AHA90294.1, ADO04239.1/E1Q7N0, ADO03869.1/E1QB24, AFF19835.1, AFF20202.1, BAJ55615.1, BAJ55257.1, BAJ56774.1, BAJ56409.1, BAJ57911.1, BAJ58250.1, BAJ59748.1, BAJ60112.1, ACI27373.1/B5Z724, ACI27771.1/B5Z872, ADU81508.1, ADU81895.1, ABF84703.1/Q1CTL9, ABF85080.1/Q1CSJ2, AFI07382.1, AFI07690.1, ADU83456.1, ADU83076.1, BAM96573.1, BAM96944.1, BAM98073.1, BAM98401.1, ACJ07817.1/B6JLN9, ACJ08194.1/B6JMR6, AFI02397.1, AFI02795.1, ADO07257.1/E1Q309, ADO06937.1/E1Q6J4, AEN15635.1, AEN15311.1, AEN18764.1, AEN18341.1, ADO05746.1/E1PWN1, ADO05445.1/E1PVT0, AFI00953.1, AFI01260.1, AFH99730.1, AFH98149.1, AFH97781.1, ACD48158.1/B2UTH6, ACD48513.1/B2UUI1, ADO02255.1/E1PZB4, ADO02630.1/E1Q0D9, AEN16830.1, ADU84619.1, ADU85018.1, AGL70321.1, AGL71396.1, AGL71039.1, ADI34738.1/D6XPP4, AFJ81975.1, AFJ81683.1	
aDGalp	1-3	aDGlcpN	GT8: AFX91429.1, CAX29588.1/C7BZI5	
aDGalp	1-3	aDGalp	GT8: AFX91429.1, CAX29588.1/C7BZI5	
aDGalp	1-3	bDGalp	GT8: AFX91429.1, CAX29588.1/C7BZI5	
aDGalp	1-4	bDGlcpN		
aDGalp	1-6	bDGlcpN		
aDGalp	1-7	aDDmanHepp		
bDGalp	1-3	aDGlcp	GT25: AEZ55696.1; GT8: AFX91429.1, CAX29588.1/C7BZI5; GT2: ADZ50785.1, ADN79242.1/E1S5Y5, AHA87806.1, AHA89378.1	
bDGalp	1-3	bDGalp	GT25: AEZ55696.1; GT8: AFX91429.1, CAX29588.1/C7BZI5; GT2: ADZ50785.1, ADN79242.1/E1S5Y5, AHA87806.1, AHA89378.1	
bDGalp	1-3	aDDmanHepp	GT25: AEZ55696.1; GT8: AFX91429.1, CAX29588.1/C7BZI5; GT2: ADZ50785.1, ADN79242.1/E1S5Y5, AHA87806.1, AHA89378.1	
bDGalp	1-3	bDGlcpN	GT25: AEZ55696.1; GT8: AFX91429.1, CAX29588.1/C7BZI5; GT2: ADZ50785.1, ADN79242.1/E1S5Y5, AHA87806.1, AHA89378.1	
bDGalp	1-3	bDGlcN
(assumed bDGlcpN)	GT25: AEZ55696.1; GT8: AFX91429.1, CAX29588.1/C7BZI5; GT2: ADZ50785.1, ADN79242.1/E1S5Y5, AHA87806.1, AHA89378.1	
bDGalp	1-3	DGlcN	GT25: AEZ55696.1; GT8: AFX91429.1, CAX29588.1/C7BZI5; GT2: ADZ50785.1, ADN79242.1/E1S5Y5, AHA87806.1, AHA89378.1	
bDGalp	1-4	bDManp	GT25: AAD07876.1/O25500, BAA88524/Q9RHG8, ADZ49888.1, ADZ51512.1, ADZ51489.1, ADZ51302.1, ACX97709.1, ADN79945.1, ADN79756.1, AHA88475.1, AHA88454.1, AHA88269.1, AHA90027.1, AHA90048.1, AHA89842.1, ACI27540.1/B5Z7J1, ABF84879.1/Q1CT43, ADU79743.1, ADU83244.1, ACJ07985.1/B6JM57, ADU84792.1; GT2: ADZ50785.1, ADN79242.1/E1S5Y5	
bDGalp	1-4	bDGlcpN	GT25: AAD07876.1/O25500, BAA88524/Q9RHG8, ADZ49888.1, ADZ51512.1, ADZ51489.1, ADZ51302.1, ACX97709.1, ADN79945.1, ADN79756.1, AHA88475.1, AHA88454.1, AHA88269.1, AHA90027.1, AHA90048.1, AHA89842.1, ACI27540.1/B5Z7J1, ABF84879.1/Q1CT43, ADU79743.1, ADU83244.1, ACJ07985.1/B6JM57, ADU84792.1; GT2: ADZ50785.1, ADN79242.1/E1S5Y5	
bDGalp	1-4	bDGlcN
(assumed bDGlcpN)	GT25: AAD07876.1/O25500, BAA88524/Q9RHG8, ADZ49888.1, ADZ51512.1, ADZ51489.1, ADZ51302.1, ACX97709.1, ADN79945.1, ADN79756.1, AHA88475.1, AHA88454.1, AHA88269.1, AHA90027.1, AHA90048.1, AHA89842.1, ACI27540.1/B5Z7J1, ABF84879.1/Q1CT43, ADU79743.1, ADU83244.1, ACJ07985.1/B6JM57, ADU84792.1; GT2: ADZ50785.1, ADN79242.1/E1S5Y5	
bDGalp	1-4	DGlcpN	GT25: AAD07876.1/O25500, BAA88524/Q9RHG8, ADZ49888.1, ADZ51512.1, ADZ51489.1, ADZ51302.1, ACX97709.1, ADN79945.1, ADN79756.1, AHA88475.1, AHA88454.1, AHA88269.1, AHA90027.1, AHA90048.1, AHA89842.1, ACI27540.1/B5Z7J1, ABF84879.1/Q1CT43, ADU79743.1, ADU83244.1, ACJ07985.1/B6JM57, ADU84792.1; GT2: ADZ50785.1, ADN79242.1/E1S5Y5	
bDGalp	1-4	DGlcN	GT25: AAD07876.1/O25500, BAA88524/Q9RHG8, ADZ49888.1, ADZ51512.1, ADZ51489.1, ADZ51302.1, ACX97709.1, ADN79945.1, ADN79756.1, AHA88475.1, AHA88454.1, AHA88269.1, AHA90027.1, AHA90048.1, AHA89842.1, ACI27540.1/B5Z7J1, ABF84879.1/Q1CT43, ADU79743.1, ADU83244.1, ACJ07985.1/B6JM57, ADU84792.1; GT2: ADZ50785.1, ADN79242.1/E1S5Y5	
bDGalp	1-4	DGlcp	GT25: AAD07876.1/O25500, BAA88524/Q9RHG8, ADZ49888.1, ADZ51512.1, ADZ51489.1, ADZ51302.1, ACX97709.1, ADN79945.1, ADN79756.1, AHA88475.1, AHA88454.1, AHA88269.1, AHA90027.1, AHA90048.1, AHA89842.1, ACI27540.1/B5Z7J1, ABF84879.1/Q1CT43, ADU79743.1, ADU83244.1, ACJ07985.1/B6JM57, ADU84792.1; GT2: ADZ50785.1, ADN79242.1/E1S5Y5	
bDGalp	1-4	DGlc	GT25: AAD07876.1/O25500, BAA88524/Q9RHG8, ADZ49888.1, ADZ51512.1, ADZ51489.1, ADZ51302.1, ACX97709.1, ADN79945.1, ADN79756.1, AHA88475.1, AHA88454.1, AHA88269.1, AHA90027.1, AHA90048.1, AHA89842.1, ACI27540.1/B5Z7J1, ABF84879.1/Q1CT43, ADU79743.1, ADU83244.1, ACJ07985.1/B6JM57, ADU84792.1; GT2: ADZ50785.1, ADN79242.1/E1S5Y5	
bDGalp	1-6	DGlcN		
bDGalp	1-7	aDDmanHepp		
bDGal	1-3	bDGlcpN	GT25: AEZ55696.1; GT8: AFX91429.1, CAX29588.1/C7BZI5; GT2: ADZ50785.1, ADN79242.1/E1S5Y5, AHA87806.1, AHA89378.1	
bDGal	1-4	bDGlcpN	GT25: AAD07876.1/O25500, BAA88524/Q9RHG8, ADZ49888.1, ADZ51512.1, ADZ51489.1, ADZ51302.1, ACX97709.1, ADN79945.1, ADN79756.1, AHA88475.1, AHA88454.1, AHA88269.1, AHA90027.1, AHA90048.1, AHA89842.1, ACI27540.1/B5Z7J1, ABF84879.1/Q1CT43, ADU79743.1, ADU83244.1, ACJ07985.1/B6JM57, ADU84792.1; GT2: ADZ50785.1, ADN79242.1/E1S5Y5	
bDGal	1-7	aDDmanHepp		
bGal	1-4	bGlcN	GT25: AAD07876.1/O25500, BAA88524/Q9RHG8, ADZ49888.1, ADZ51512.1, ADZ51489.1, ADZ51302.1, ACX97709.1, ADN79945.1, ADN79756.1, AHA88475.1, AHA88454.1, AHA88269.1, AHA90027.1, AHA90048.1, AHA89842.1, ACI27540.1/B5Z7J1, ABF84879.1/Q1CT43, ADU79743.1, ADU83244.1, ACJ07985.1/B6JM57, ADU84792.1; GT2: ADZ50785.1, ADN79242.1/E1S5Y5	
Galp	1-6	bDGlcpN		
bDGalpN	1-3	bDGalp		
aDGalpN	1-3	bDGalp		
aDGalpN	1-3	bDGal		
aDGlcp	1-2	aDGlcp	GT8: AAD08459.1/O25962, AJF08471.1, AJF09629.1, AJF08520.1, AJF10014.1, AJF11169.1 AJF10062.1, ACX99857.1/D0K187, AFX91737.1, AFX90576.1, AFX89074.1, AHA87859.1, AHA87479.1, AHZ28072.1, AHZ27708.1, AHZ28121.1, AHA89431.1, AHA89050.1, AHZ26321.1, AHZ26366.1, AHZ27397.1, AHZ29248.1, AHZ29292.1, AHZ30323.1, ADO04567.1/E1Q9B3, BAJ55910.1, BAJ57381.1, BAJ58884.1, BAJ60405.1, ACI28087.1/B5Z938, AJD66213.1, AJD65210.1, AJD65251.1, ABF85409.1/Q1CRL3, AHZ25659.1, AHZ24873.1, ADU82612.1, BAO97406.1, BAM98692.1, AHN34228.1, AHN34088.1, AHN34873.1, AHN36353.1, AHN35566.1, AHN38198.1, AHN37145.1, AHN38541.1, AHN39615.1, AHN40010.1, AHN40655.1, AHN39876.1, AHN42516.1, AHN41449.1, AHN42882.1, AHN43958.1, AHN44365.1, AHN45019.1, AHN44226.1, ACJ08536.1/B6JNQ8, ADO07584.1/E1Q4F6, AEN15962.1, AEN19091.1, AFV44232.1, AFV42974.1, AFV44569.1, AFV45825.1, ADO06058.1/E1PXV3, AFI01614.1, AFI00047.1, AFH98491.1, ACD48839.1/B2UVF7, AGR62474.1, AGR62757.1, AGR63292.1, AGL68348.1, AGL68727.1, AGL68301.1, AEN17521.1, AEN16369.1, AGL66623.1, AGL66243.1, AGL66196.1, AGL69233.1, AGL70471.1, AGL70732.1, AGL71913.1, ADI34271.1/D6XNC7, AFJ82253.1	
aDGlcp	1-2	aDDmanHepp	GT8: AAD08459.1/O25962, AJF08471.1, AJF09629.1, AJF08520.1, AJF10014.1, AJF11169.1 AJF10062.1, ACX99857.1/D0K187, AFX91737.1, AFX90576.1, AFX89074.1, AHA87859.1, AHA87479.1, AHZ28072.1, AHZ27708.1, AHZ28121.1, AHA89431.1, AHA89050.1, AHZ26321.1, AHZ26366.1, AHZ27397.1, AHZ29248.1, AHZ29292.1, AHZ30323.1, ADO04567.1/E1Q9B3, BAJ55910.1, BAJ57381.1, BAJ58884.1, BAJ60405.1, ACI28087.1/B5Z938, AJD66213.1, AJD65210.1, AJD65251.1, ABF85409.1/Q1CRL3, AHZ25659.1, AHZ24873.1, ADU82612.1, BAO97406.1, BAM98692.1, AHN34228.1, AHN34088.1, AHN34873.1, AHN36353.1, AHN35566.1, AHN38198.1, AHN37145.1, AHN38541.1, AHN39615.1, AHN40010.1, AHN40655.1, AHN39876.1, AHN42516.1, AHN41449.1, AHN42882.1, AHN43958.1, AHN44365.1, AHN45019.1, AHN44226.1, ACJ08536.1/B6JNQ8, ADO07584.1/E1Q4F6, AEN15962.1, AEN19091.1, AFV44232.1, AFV42974.1, AFV44569.1, AFV45825.1, ADO06058.1/E1PXV3, AFI01614.1, AFI00047.1, AFH98491.1, ACD48839.1/B2UVF7, AGR62474.1, AGR62757.1, AGR63292.1, AGL68348.1, AGL68727.1, AGL68301.1, AEN17521.1, AEN16369.1, AGL66623.1, AGL66243.1, AGL66196.1, AGL69233.1, AGL70471.1, AGL70732.1, AGL71913.1, ADI34271.1/D6XNC7, AFJ82253.1	
aDGlcp	1-3	aDGlcp		
aDGlcp	1-3	aDGlcpA		
aDGlcp	1-3	aDGalp		
aDGlcp	1-4	bDGalp	GT8: AAD08459.1/O25962	
aDGlcp	1-4	aDGlcp	GT8: AAD08459.1/O25962	
aDGlcp	1-4	aDGalp	GT8: AAD08459.1/O25962	
aDGlcp	1-4	bDGal	GT8: AAD08459.1/O25962	
aDGlcp	1-6	aDDmanHepp	GT8: AAD07221.1/O24967, AAD05728.1/Q9ZMS1, BAM96075.1, BAM97594.1	
aDGlcp	1-6	aDGlcp	GT8: AAD07221.1/O24967, AAD05728.1/Q9ZMS1, BAM96075.1, BAM97594.1	
aDGlcp	1-6	bDGlcpN	GT8: AAD07221.1/O24967, AAD05728.1/Q9ZMS1, BAM96075.1, BAM97594.1	
aDGlcp	1-7	aDDmanHepp		
aDGlcp	1-1	Gro		
aDGlc	1-4	bDGal	AAD08459.1/O25962 (GT8)	
aDGlcpA	1-4	aDGlcp		
aDGlcpN	1-7	aDDmanHepp		
bDGlcpN	1-2	bDRibf		
bDGlcpN	1-2	aDDmanHepp		
bDGlcpN	1-3	aDDmanHepp	GT8: AAD06608.1/Q9ZKA7, AAD08151.1/O25733, BAM96973.1, BAM96974.1, BAM98430.1, BAM98429.1	
bDGlcpN	1-3	bDGalp	GT8: AAD06608.1/Q9ZKA7, AAD08151.1/O25733, BAM96973.1, BAM96974.1, BAM98430.1, BAM98429.1	
bDGlcpN	1-3	bDGal	GT8: AAD06608.1/Q9ZKA7, AAD08151.1/O25733, BAM96973.1, BAM96974.1, BAM98430.1, BAM98429.1	
bDGlcpN	1-4	DGlcN		
bDGlcpN	1-6	aDGlcpN		
bDGlcpN	1-6	aDDmanHepp		
bDGlcpN	1-7	aDDmanHepp		
bDGlcpN	1-9	9HONno		
bDGlcpN	1-?	aDDmanHepp		
bDGlcN
(assumed bDGlcpN)	1-3	bDGalp	GT8: AAD06608.1/Q9ZKA7, AAD08151.1/O25733, BAM96973.1, BAM96974.1, BAM98430.1, BAM98429.1	
bGlcN	1-3	bGal	GT8: AAD06608.1/Q9ZKA7, AAD08151.1/O25733, BAM96973.1, BAM96974.1, BAM98430.1, BAM98429.1	
aNeup	2-2	bDGalp	GT_NC: ADZ49418.1, ADZ51019.1, AFV41551.1, BAJ58560.1, AJD65358.1, AHZ25033.1, BAM97759.1 et al. (63 enzymes from the GT_NC family possible involved in transfer of neuraminic acid to the coat polysaccharide)	
aNeup	2-3	bDGalp	GT_NC: ADZ49418.1, ADZ51019.1, AFV41551.1, BAJ58560.1, AJD65358.1, AHZ25033.1, BAM97759.1 et al. (63 enzymes possible involved in transfer of neuraminic acid to the coat polysaccharide); GT42: AFX89576.1, AFX89577.1, ADO04052.1/E1Q743, ADO04054.1/E1Q745, AEN15449.1, AEN18521.1, AEN18522.1, ADO05253.1/E1PV88, ADO05254.1/E1PV89, AFH99551.1, AFH99552.1, AFH97957.1, AFH97958.1	
aNeup	2-6	bDGalp	GT_NC: ADZ49418.1, ADZ51019.1, AFV41551.1, BAJ58560.1, AJD65358.1, AHZ25033.1, BAM97759.1 et al. (63 enzymes from the GT_NC family possible involved in transfer of neuraminic acid to the coat polysaccharide)	
aDManp	1-2	aDManp		
aDManp	1-3	aLRhap		
aDManp	1-3	aDGlcp		
aDManp	1-3	aDManp		
aDManp	1-6	aDManp		
aDRhap	1-2	aDManp3CMe		
aDRhap	1-2	aDManp		
aDRhap	1-3	D1dEry-ol		
aLRhap	1-3	aDRhap		
bDRibf	1-2	bDRibf		
bDRibf	1-2	DThre-ol		
bDRibf	1-4	bDGalp		
aKdop	2-6	bDGlcpN		
aKdo	2-6	bDGlcpN		
aDManp3CMe	1-3	aLRhap		
* Based on information obtained from CSDB and the CAZy database in March 2015. Disaccharides were obtained by using the Fragment abundance tool from the BCSDB database with the following parameters: species = Helicobacter pylori; anomeric forms are not combined; undefined configs, aglycons in oligomers, and aliases are included; 'Subst' aliases are explained. GenBank and UniProt IDs were obtained from the CAZy database manually by searching the database for GTs assigned to Helicobacter pylori. The following dimeric fragments were not inlcuded: with underdetermined linkage positions; containing an explained alias implying a superclass of residues; containing phosphoric or sulfuric acid residue; containing hexitols, which are likely analytical artifacts. 
** Characterized GTs are shown in black, uncharacterized/predicted ones are shown in dark red; the GT family names are also indicated. Residues with undetermined absolute, anomeric or ringsize configurations are grayed. Residue names are in CSDB notation.

Table S2a. Part of Table S2 (not included in the main table body) containing disaccharides with an ambiguous acceptor or a superclass acceptor, with phosphate/sulphate residues or with supposed analytical artifacts (hexitols). 
Donor	Linkage	Acceptor	Glycosyltranferases (GenBank/UniProt)	
aLFucp	1-3	anhMan-ol	GT10: AAB93985.1/O32631, AAD06169.1/Q9ZLI3, AAD06573.1/Q9ZKD7, AAB81031.1/O30511, AAF35291.2/Q9L8S4, ABZ68520.1, ADZ49735.1, ADZ51338.1, AAD07447.1/O25142, AAD07710.1/O25366, ACX97875.1/D0ISI2, ACX98174.1/D0ITD1, ACX99573.1/D0K0F3, ADN79791.1/E1S8G1, ADN80190.1/E1S9L0, AFX91028.1, AFX91400.1, AFX89961.1, AFX90099.1, CAX29250.1/C7BZU7, CAX29559.1/C7BXF2, CBI65984.1/D7FCS7, AHA88301.1, AHA88720.1, AHA89874.1, AHA90294.1, ADO04239.1/E1Q7N0, ADO03869.1/E1QB24, AFF19835.1, AFF20202.1, BAJ55615.1, BAJ55257.1, BAJ56774.1, BAJ56409.1, BAJ57911.1, BAJ58250.1, BAJ59748.1, BAJ60112.1, ACI27373.1/B5Z724, ACI27771.1/B5Z872, ADU81508.1, ADU81895.1, ABF84703.1/Q1CTL9, ABF85080.1/Q1CSJ2, AFI07382.1, AFI07690.1, ADU83456.1, ADU83076.1, BAM96573.1, BAM96944.1, BAM98073.1, BAM98401.1, ACJ07817.1/B6JLN9, ACJ08194.1/B6JMR6, AFI02397.1, AFI02795.1, ADO07257.1/E1Q309, ADO06937.1/E1Q6J4, AEN15635.1, AEN15311.1, AEN18764.1, AEN18341.1, ADO05746.1/E1PWN1, ADO05445.1/E1PVT0, AFI00953.1, AFI01260.1, AFH99730.1, AFH98149.1, AFH97781.1, ACD48158.1/B2UTH6, ACD48513.1/B2UUI1, ADO02255.1/E1PZB4, ADO02630.1/E1Q0D9, AEN16830.1, ADU84619.1, ADU85018.1, AGL70321.1, AGL71396.1, AGL71039.1, ADI34738.1/D6XPP4, AFJ81975.1, AFJ81683.1	
bDGalp	1-4	anhMan-ol	GT25: AAD07876.1/O25500, BAA88524/Q9RHG8, ADZ49888.1, ADZ51512.1, ADZ51489.1, ADZ51302.1, ACX97709.1, ADN79945.1, ADN79756.1, AHA88475.1, AHA88454.1, AHA88269.1, AHA90027.1, AHA90048.1, AHA89842.1, ACI27540.1/B5Z7J1, ABF84879.1/Q1CT43, ADU79743.1, ADU83244.1, ACJ07985.1/B6JM57, ADU84792.1; GT2: ADZ50785.1, ADN79242.1/E1S5Y5	
bDGalp	1-4	O-chain-core		
aDGlcp	1-6	-6)aDGlcp(1-	GT8: AAD07221.1/O24967, AAD05728.1/Q9ZMS1, BAM96075.1, BAM97594.1	
aDGlcp	1-6	1,6-a-D-glucan		
bDGlcN	1-3	DGal-ol	GT8: AAD06608.1/Q9ZKA7, AAD08151.1/O25733, BAM96973.1, BAM96974.1, BAM98430.1, BAM98429.1	
bDGlcpN	1-6	DGlcN-ol		
bDGlcpN	1-3	DGal-ol		
bDGlcpN	1-3	O-antigen		
bDGlcpN	1-3	O-chain		
aKdop	2-6	anhMan-ol		
aDDmanHepp	1-3	O-chain-heptan	GT9: AAX12679.1/Q5D6B0, ADZ50261.1, ADZ49368.1, ADZ51869.1, ADZ50969.1, AAD07342.1/O25056, AAD08237.1/O25802, AJF09417.1, AJF09505.1, AJF10958.1, AJF11046.1, ADU41556.1, ADU40654.1, ADU41461.1, ACX97491.1/D0IRE8, ACX98281.1/D0ITN8, ACX98900.1/D0JYI0, ACX99679.1/D0K0Q9, ACX99065.1/D0JYZ5, AEE70903.1, AEE70807.1, AEE69937.1, ADN79423.1/E1S7E3, ADN80404.1/E1S614, ADN80317.1/E1S9Y7, AFX91513.1, AFX90693.1, AFX89301.1, CAX28829.1/C7BYL9, CAX29815.1/C7C096, CBI65857.1/D7FCF0, CBI66840.1/D7FF83, AHA87976.1, AHA87527.1, AHA87687.1, AHZ27755.1, AHZ27907.1, AHA89259.1, AHA89548.1, AHA89098.1, AHZ27290.1, ADO04360.1/E1Q852, ADO03520.1/E1Q9P4, AFF20600.1, AFF20758.1, BAJ55726.1, BAJ54883.1, BAJ56298.1, BAJ57114.1, BAJ57870.1, BAJ58706.1, BAJ60228.1, BAJ59406.1, ACI27025.1/B5ZA45, ACI27886.1/B5Z8I7, ACI27201.1/B5Z6K2, ADU82017.1, ADU81160.1, AJD66044.1, AJD66128.1, ABF84348.1/Q1CUM4, ABF85199.1/Q1CS73, ABF84523.1/Q1CU49, AFI06987.1, AFI07806.1, ADU79509.1, ADU80449.1, AHZ25937.1, AHZ25850.1, AAD05843.1/Q9ZMF6, AAD06697.1/Q9ZK26, AAB65778.1/O30355, ADU82729.1, ADU83580.1, BAO97204.1, BAO97917.1, BAO97297.1, ADU82904.1, AAX12681.1/Q5D6A8, BAM96196.1, BAM97057.1, BAM98515.1, BAM97711.1, AHN35146.1, AHN36622.1, AHN37987.1, AHN39429.1, AHN40921.1, AHN42327.1, AHN43769.1, AHN45294.1, ACJ08309.1/B6JN31, ACJ07436.1/B6JKK8, AFI02069.1, AFI02915.1, ADO07374.1/E1Q3C6, ADO06530.1/E1Q539, AAX12680.1/Q5D6A9, AEN14924.1, AEN15757.1, AEN17991.1, AEN18885.1, AFV43096.1, AFV44002.1, AFV44690.1, AFV45595.1, ADO05028.1/E1PUI1, ADO05860.1/E1PWZ5, AFI01376.1, AFI00560.1, AFH99016.1, AFH99846.1, AFH98291.1, AFH97437.1, ACD48635.1/B2UUV3, ACD47742.1/B2USB0, ADO01898.1/E1PXZ9, ADO02755.1/E1Q0R4, AGL70824.1, AGL70916.1, AEN16487.1, AEN17320.1, AGT73551.1, AGT74495.1, ADU85141.1, ADU84307.1, AGL66809.1, AGL66716.1, AGL69452.1, AGL69358.1, AGL65939.1, AGR62916.1, AGR62833.1, AGL68913.1, AGL68821.1, ADI34388.1/D6XNP4, ADI35260.1/D6XR66, AFJ82089.1, FJ81284.1	
aDDmanHepp	1-?	aDDmanHepp		
O-chain-heptan (-3-a-DDHep, -2-a-DDHep, -6-a-DDHep)	1-7	aDDmanHepp		
O-chain-heptan	1-3	aDDmanHepp		
O-chain-heptan	1-7	aDDmanHepp		
aDGlcpN	1-0	P		
aDFucp	1-0	P		
bLFucp	1-0	P		
aLFucpN4N	1-0	P		
aDGalp	1-0	P		
bDGalp	1-0	S		
bDGlcpN	1-3	O-chain region	GT8: AAD06608.1/Q9ZKA7, AAD08151.1/O25733, BAM96973.1, BAM96974.1, BAM98430.1, BAM98429.1	
bDGlcpN	1-3	O-antigen(Lex)-core-lipid A		
bDGlcpN	1-3	O-antigen-core-lipid A		
bDGlcpN	1-3	O-antigen-heptan-core-lipid A		
bDGlcpN	1-4	polysaccharide chain		
aDRhap	1-0	P		
bLRhap	1-0	P		
bL6daraHexp-4-ulo	1-0	P		
bL6daraHexpN-4-ulo	1-0	P		
aD6dlyxHexp-4-ulo	1-0	P		
bL6dlyxHexp-4-ulo	1-0	P		
bL6dlyxHexpN-4-ulo	1-0	P		
aD6dxylHexp-4-ulo	1-0	P		
aD6dxylHexpN-4-ulo	1-0	P		
bLArap4N	1-0	P		
bLQuip	1-0	P		
aDQuipN4N	1-0	P		
bL6dAltpN4N	1-0	P		
aPsep	2-0	P		
LthrPen-4-ulo	1-0	P		
aKdop	2-6	lipid A		


Table S3. Disaccharides and predicted glycosyltransferases found in Neisseria gonorrhoeae, Neisseria meningitides, and Staphylococcus aureus*,a
Dimer	Neisseria gonorrhoeae**	Neisseria meningitidis**	Staphylococcus aureus**	
aDGalp(1-3)bDGalp	√	√
GT4: CBA05071.1; GT97: CCP19949.1		
aDGalp(1-4)bDGalp	√
GT8: AAA68011.1	√
GT4: CAA74303.1, CBA05071.1; GT8: AAB48385.1, AAK85137.1, AAK85142.1, AAK85146.1, ADZ02865.1; GT97: CAA74303.1; GT97: CCP19949.1		
bDGalp(1-4)aDGalp	√
GT25: AAA68010.1, AAA68013.1	√
GT4: CBA05071.1; GT25: AAX58721.1, AAK85139.1; GT97: CCP19949.1		
bDGalp(1-4)bDGalp	√
GT25: AAA68010.1, AAA68013.1	√
GT4: CBA05071.1; GT25: AAX58721.1, AAK85139.1; GT97: CCP19949.1		
bDGalp(1-4)bDGlcp	√
GT25: AAA68010.1, AAA68013.1	√
GT4: CBA05071.1; GT25: AAX58721.1, AAK85139.1; GT97: CCP19949.1		
bDGalp(1-4)bDGlcpN	√
GT25: AAA68010.1, AAA68013.1	√
GT4: CBA05071.1; GT25: AAX58721.1, AAK85139.1; GT97: CCP19949.1		
bDGalpN(1-3)bDGalp	√
GT2: AAA68012.1	√		
aDGlcp(1-3)aXLDmanHepp	√
GT4: ACF31133.1, ACF31134.1	√
GT4: CAA74302.1, AAM33535.1, AAM33533.1, AAM33534.1; GT8: ADO32346.1; GT97: CAA74302.1		
bDGlcp(1-3)aXLDmanHepp	√
GT4: ACF31133.1, ACF31134.1	√
GT8: ADO32346.1		
bDGlcp(1-4)aXLDmanHepp	√
GT2: AAW90002.1, EEZ48399.1, ACF31133.1, ACF31134.1	√
GT2: AAC44647.1, ABX73762.1, AHW76474.1, CAX49571.1, CCA44113.1, ADO32142.1, AAC44647.1, CAM10809.1, ADY94254.1, ADY95157.1, AJC63756.1, ADY97097.1, ADZ00116.1, ADZ01036.1, AIZ25724.1, AIZ21040.1, AAF42052.1, AIZ22925.1, AIZ24893.1, AIZ18716.1, ADZ04074.1, CBY91348.1; GT8: ADO32346.1		
bDGlcp(1-4)bDGlcp	√
GT2: AAW90002.1, EEZ48399.1, ACF31133.1, ACF31134.1	√
GT2: AAC44647.1, ABX73762.1, AHW76474.1, CAX49571.1, CCA44113.1, ADO32142.1, AAC44647.1, CAM10809.1, ADY94254.1, ADY95157.1, AJC63756.1, ADY97097.1, ADZ00116.1, ADZ01036.1, AIZ25724.1, AIZ21040.1, AAF42052.1, AIZ22925.1, AIZ24893.1, AIZ18716.1, ADZ04074.1, CBY91348.1; GT8: ADO32346.1		
aDGlcpN(1-2)aXLDmanHepp	√
GT4: AAW90003.1, EEZ48398.1	√
GT4: AAC44648.1, AAF42053.1, ABX73763.1, AHW76475.1, CAX49570.1, ADO32143.1, CAM10810.1, ADY94255.1, ADY95156.1, ADY97096.1, ADZ00117.1, ADZ01035.1, AIZ25725.1, AIZ21041.1, AIZ22926.1, AIZ18715.1, ADZ04075.1, CBY91349.1		
bDGlcpN(1-3)bDGalp	√
GT2: AAA92074.1; GT4: EEZ48398.1	√
GT2: AAC44084.1, AAK85138.1, AAK85143.1, AAK85147.1, ABC00912.1, ADY94255.1, ADY95156.1, ADY97096.1, ADZ00117.1, ADZ01035.1, ADZ04075.1		
aXLDmanHepp(1-3)aXLDmanHepp	√
GT9: AAA93059.1, AAA72092.1, AAW90547.1, CAA85504.1, EEZ49039.1, EEZ47579.1, ACF29483.1, ACF30921.1	√
GT9: AIZ20882.1, CCA45828.1, ADO31991.1, ADO32556.1, CAM10663.1, CAM11282.1, ADY94104.1, ADY94686.1, ADY96647.1, ADY95326.1, AJC63910.1, ADY98583.1, ADY97237.1, ADY99957.1, ADZ00555.1, ADZ01178.1, AIZ25568.1, AIZ20882.1, CAV30631.1, AAA79139.1, ABX73600.1, ABX74232.1, AHW76227.1, AHW74579.1, CAX49745.1, CBA03345.1, CBA06870.1, CBA08605.1, CBA06859.1, CBA06378.1, CBA05514.1, CBY91140.1, AAF42464.1, AAF41882.1, AIZ22765.1, AIZ24732.1, AIZ18877.1, ADZ04450.1, ADZ03917.1		
aXLDmanHepp(1-5)aXKdop	√
GT9: AAA93059.1, AAA72092.1, AAW90547.1, CAA85504.1, EEZ49039.1, EEZ47579.1, ACF29483.1, ACF30921.1	√
GT9: AAA86622.1, CCA45828.1, ADO31991.1, ADO32556.1, CAM10663.1, CAM11282.1, ADY94104.1, ADY94686.1, ADY96647.1, ADY95326.1, AJC63910.1, ADY98583.1, ADY97237.1, ADY99957.1, ADZ00555.1, ADZ01178.1, AIZ25568.1, AIZ20882.1, CAV30631.1, AAA79139.1, ABX73600.1, ABX74232.1, AHW76227.1, AHW74579.1, CAX49745.1, CAX51114.1, CBA03345.1, CBA06870.1, CBA08605.1, CBA06859.1, CBA06378.1, CBA05514.1, CBY91140.1, AAF42464.1, AAF41882.1, AIZ22765.1, AIZ24732.1, AIZ18877.1, ADZ04450.1, ADZ03917.1, CBY91887.1		
aXKdop(2-4)aXKdop	√
GT30: AAW90528.1, EEZ49058.2, ACF30942.1	√
GT30: ABX72246.1, AHW74602.1, CAX49006.1, CBA03305.1, CBA08540.1, CCA43556.1, ADO30560.1, CAM11300.1, ADY92776.1, ADY94704.1, AJC63297.1, ADY96666.1, ADY98601.1, ADZ00573.1, AIZ26207.1, AIZ19370.1, AAF40493.1, AIZ23039.1, AIZ25381.1, AIZ18222.1, ADZ02521.1, CBY89835.1		
bDGlcpN(1-6)bDGlcpN		√
GT2: AAC44084.1; GT4: ADY94255.1, ADY95156.1, ADY97096.1, ADZ00117.1, ADZ01035.1, ADZ04075.1	√
GT2: AAD52055.1, AFR74676.1, AGP29609.1, AGO31035.1, AFQ91281.1, AFQ91282.1, AHW68324.1, AEW66623.1, AFH69005.1, AFH70942.1, CEH27485.1, AGU62808.1, AAW38686.1, ADI99155.1, ACY12568.1, AIW28333.1, AIU86725.1, CCG17280.1, ADL24476.1, CCC89204.1, CAG41723.1, CCE60320.1, CAG44369.1, BAB96451.1, BAB43764.1, ABD31989.1, CCJ11910.1, ADL66719.1, CBI50667.1, ABD22205.1, ABX30652.1, BAF68837.1, ADQ75982.1, AHJ08470.1, AID41380.1	
bDGalp(1-4)aDGlcp	√
GT25: AAA68010.1, AAA68013.1			
aDGalpN(1-3)bDQuipN4N	√
GT2: AAA68012.1			
bDGalpN(1-4)bDGalp	√
GT2: AAA68012.1			
aXKdop(2-8)aXKdop	√
GT30: AAW90528.1, EEZ49058.2, ACF30942.1			
aDGalp(1-4)bDGlcp		√
GT4: CAA74303.1, CBA05071.1; GT8: AAB48385.1, AAK85137.1, AAK85142.1, AAK85146.1, ADZ02865.1; GT97: CCP19949.1		
aDGalp(1-4)aXNeup		√
GT4: CAA74303.1, CBA05071.1; GT8: AAB48385.1, AAK85137.1, AAK85142.1, AAK85146.1, ADZ02865.1; GT97: CCP19949.1		
bDGalp(1-3)aDGlcpN		√
GT4: CBA05071.1; GT97: CCP19949.1		
bDGalp(1-4)aXLDmanHepp		√
GT4: CBA05071.1; GT25: AAX58721.1, AAK85139.1; GT97: CCP19949.1		
aDGalpN(1-3)bDGalp		√		
bDGalpN(1-3)aDGalp		√		
bDGlcp(1-2)aXLDmanHepp		√
GT8: ADO32346.1		
bDGlcp(1-4)bDGlcpN		√
GT2: AAC44647.1, ABX73762.1, AHW76474.1, CAX49571.1, CCA44113.1, ADO32142.1, AAC44647.1, CAM10809.1, ADY94254.1, ADY95157.1, AJC63756.1, ADY97097.1, ADZ00116.1, ADZ01036.1, AIZ25724.1, AIZ21040.1, AAF42052.1, AIZ22925.1, AIZ24893.1, AIZ18716.1, ADZ04074.1, CBY91348.1; GT8: ADO32346.1		
aDGlcpN(1-3)bDGalp		√
GT4: ADY94255.1, ADY95156.1, ADY97096.1, ADZ00117.1, ADZ01035.1, ADZ04075.1		
bDGlcpN(1-3)aDGlcpN		√
GT2: AAC44084.1, AAK85138.1, AAK85143.1, AAK85147.1, ABC00912.1; GT4: ADY94255.1, ADY95156.1, ADY97096.1, ADZ00117.1, ADZ01035.1, ADZ04075.1		
bDGlcpN(1-3)bDGlcpN		√
GT2: AAC44084.1, AAK85138.1, AAK85143.1, AAK85147.1, ABC00912.1; GT4: ADY94255.1, ADY95156.1, ADY97096.1, ADZ00117.1, ADZ01035.1, ADZ04075.1		
bDGlcpN(1-6)aDGlcpN		√
GT2: AAC44084.1; GT4: ADY94255.1, ADY95156.1, ADY97096.1, ADZ00117.1, ADZ01035.1, ADZ04075.1		
aLRhap(1-2)aLRhap		√		
aLRhap(1-5)aXKdop		√		
aXLDmanHepp(1-2)aXLDmanHepp		√
GT9: AAC44079.1, CCA45828.1, ADO31991.1, ADO32556.1, CAM10663.1, CAM11282.1, ADY94104.1, ADY94686.1, ADY96647.1, ADY95326.1, AJC63910.1, ADY98583.1, ADY97237.1, ADY99957.1, ADZ00555.1, ADZ01178.1, AIZ25568.1, AIZ20882.1, CAV30631.1, AAA79139.1, ABX73600.1, ABX74232.1, AHW76227.1, AHW74579.1, CAX49745.1, CBA03345.1, CBA06870.1, CBA08605.1, CBA06859.1, CBA06378.1, CBA05514.1, CBY91140.1, AAC44079.1, AAF42464.1, AAF41882.1, AIZ22765.1, AIZ24732.1, AIZ18877.1, ADZ04450.1, ADZ03917.1		
aXKdop(2-6)bDGlcpN		√
GT30: ABX72246.1, AHW74602.1, CAX49006.1, CBA03305.1, CBA08540.1, CCA43556.1, ADO30560.1, CAM11300.1, ADY92776.1, ADY94704.1, AJC63297.1, ADY96666.1, ADY98601.1, ADZ00573.1, AIZ26207.1, AIZ19370.1, AAF40493.1, AIZ23039.1, AIZ25381.1, AIZ18222.1, ADZ02521.1, CBY89835.1		
aXNeup(2-3)bDGalp		√
GT4: CAA74302.1; GT52: AAC44543.1, AAC44541.1, CBA05377.1 ; GT97: CAA74302.1, ABW08129.1, CCP19949.1, CCP19896.1, CCP19868.1		
aXNeup(2-8)aXNeup		√
GT4: CAA74302.1; GT38: AAA20478.1, AAP34768.1, ADZ02565.1, ABX72299.1, CCA43626.1, AEQ61988.1, AAB53842.1, ADY92830.1, ADY94748.1, ADY96708.1, ADY98646.1, ADZ00630.1, AAO85290.1, AAP34769.1, AAP34767.1, AAO85289.1, AAP34770.1, AAO85288.1, AAP34771.1, AAO85291.1, AAP34766.1, AAP34768.1, ADZ02565.1; GT97: CAA74302.1, ABW08129.1, CCP19949.1, CCP19896.1, CCP19868.1		
aXNeup(2-9)aXNeup		√
GT4: CAA74302.1; GT38: CCP19660.1, CAX49051.1, AJC63348.1, CCP19660.1; GT97: CAA74302.1, ABW08129.1, CCP19949.1, CCP19896.1, CCP19868.1		
aDGalpNA(1-4)aDGalpNA			√	
aDGlcp(1-3)xLGro?			√
GT2: AFR72783.1, CCC87405.1, CAG39767.1, CCE58504.1, CAG42445.1, AII55223.1, AGW35690.1, AGW33158.1, CBI48658.1; GT4: ADC36753.1, ADC36752.1, ADC38798.1, ADC37201.1, CCW22971.1, CCW20892.1, CCW20893.1, CCW21273.1, CAI80202.1, CAI80203.1, CEH26167.1, CEH25321.1, CEH27465.1, CEH25320.1, CCP89500.1, EEV04375.1, EEV04374.1, AFH68862.1, AFH68863.1, AFH70922.1, AFH69255.1, AGU62789.1, AGU60693.1, AGU60692.1, AIW26660.1, AIW28314.1, AIW26228.1, AIW26227.1, ABR51459.1, ABR51460.1, ABQ48391.1, ABQ48392.1, ADL22471.1, ADL22470.1, ADL24458.1, AEV77623.1, AEV78028.1, BAB56727.1, BAB56726.1, AII55490.1, CCJ22186.1, CCJ18257.1, AJE63971.1, AJE63972.1, AJE66024.1, ADL66702.1, ADL64632.1, ADL64633.1, AGY89076.1, AGY90866.1, AGY88668.1, AGY88667.1, AID39062.1; GT8: ADL24457.1, AJE66023.1, ADL66701.1; GT28: AJP29404.1, AJP19409.1, AJP65400.1, AGP27932.1, AJP26756.1, AIL57441.1, AJC39657.1, AJC36817.1, AJC42504.1, AJC28280.1, AJC31127.1, AJC33970.1, AHM68872.1, AJP19409.1, AJP29404.1, AJP65400.1, AGP27932.1, CCW21255.1, CAI80571.1, AJP22160.1, AEW64985.1, EEV04177.1, AGU54736.1, AFH69235.1, AIO20596.1, AGU61075.1, ADI97477.1, ACY10894.1, AIU85291.1, AHZ98810.1, ADL22820.1, CCC87700.1, CCE58718.1, BAB94763.1, AII55471.1, AGW35932.1, AGW33399.1, CCJ12648.1, CAQ49440.1, AJE64422.1, ADL65019.1, BAF67159.1, AEB88099.1, CBI48893.1, ABX28995.1, AEZ37013.1, AIA27512.1, AHJ06744.1	
aDGlcp(1-6)aDGlcp			√
GT2: AFR72783.1, CCC87405.1, CAG39767.1, CCE58504.1, CAG42445.1, AII55223.1, AGW35690.1, AGW33158.1, CBI48658.1; GT8: ADL24457.1, AJE66023.1, ADL66701.1	
bDGlcp(1-2)aDGlcp			√
GT2: AFR72783.1, CCC87405.1, CAG39767.1, CCE58504.1, CAG42445.1, AII55223.1, AGW35690.1, AGW33158.1, CBI48658.1	
bDGlcp(1-2)bDGlcp			√
GT2: AFR72783.1, CCC87405.1, CAG39767.1, CCE58504.1, CAG42445.1, AII55223.1, AGW35690.1, AGW33158.1, CBI48658.1	
bDGlcp(1-6)bDGlcp			√
GT2: AFR72783.1, CCC87405.1, CAG39767.1, CCE58504.1, CAG42445.1, AII55223.1, AGW35690.1, AGW33158.1, CBI48658.1	
aDGlcpN(1-2)xDGro?			√
GT2: AFR74676.1, AFH70942.1, AGU62808.1, CCG17280.1, CCC89204.1, CAG41723.1, CCE60320.1, CAG44369.1; GT28: AFR73448.1, EEV04077.1, ADL23224.1, AJE64844.1, ADL65425.1, ABX29365.1	
bDGlcpN(1-4)bXMurp			√
GT2: AFR74676.1, AFH69005.1, AFH70942.1, AGU62808.1, CCG17280.1, CCC89204.1, CAG41723.1, CCE60320.1, CAG44369.1; GT28: AFR73448.1, EEV04077.1, ADL23224.1, AJE64844.1, ADL65425.1, ABX29365.1	
aLFucpN(1-3)bDFucpN			√
GT4: AAW37444.1, CBX33520.1, CCG14844.1, and several others predicted to be close to wbuB in E. coli 	
bXMurp(1-4)bDGlcpN			√	
* Based on information obtained from CSDB and the CAZy database in March 2015. Disaccharides were obtained by using the taxon clustering tool from the CSDB database with the following parameters: rank = species (Neisseria gonorrhoeae, Neisseria meningitides, Staphylococcus aureus); normalized taxon population threshold = 1‰; structure abundance threshold = 10; fragment abundance threshold = 11; fragment presence threshold = 1; size of fragments to analyze = two residues; type of structures to analyze = any; monovalent residues, underdetermined resides, residue superclasses, and non-sugar residues are excluded; anomeric forms were not combined; fragment location was not differentiated. GenBank IDs were obtained from the CAZy database manually by searching the database for GTs assigned to Neisseria gonorrhoeae, Neisseria meningitides, or Staphylococcus aureus.
** √ indicates the presence of a disaccharide in carbohydrate structures from a given species; characterized GTs are shown in black, uncharacterized/predicted ones are shown in dark red; the GT family names are also indicated. 

a All three species also have disaccharides to which no GT activity has been assigned yet, as well as predicted GTs for which no saccharide products have been found in CSDB:
Neisseria gonorrhoeae: alpha-2,3- and alpha-2,6-sialyltransferases were found (AAW89748.1, AAC44539.1, AAW89748.1 and other GTs from the GT52 family).
Neisseria meningitidis: â-galactosamide á-2,3-sialyltransferases (GT52) were found (e.g. AAC44544.2, ABX73045.1, CAX50303.1, CBA09022.1).
Staphylococcus aureus: beta-D-Glcp alpha-1,6-galactosyltransferases (GT4, AFH68448.1, AGU60242.1), several putative galactosyltransferases from GT4, and putative N-acetylmannosaminyltransferases (GT26) were found.
